# Diversity and function of terpene synthases in the production of carrot aroma and flavor compounds

**DOI:** 10.1038/s41598-020-66866-1

**Published:** 2020-06-19

**Authors:** Andrew Muchlinski, Mwafaq Ibdah, Shelby Ellison, Mossab Yahyaa, Bhagwat Nawade, Suzanne Laliberte, Douglas Senalik, Philipp Simon, Susan R. Whitehead, Dorothea Tholl

**Affiliations:** 1 Department of Biological Sciences, Virginia Tech, 24061 Blacksburg, Virginia USA; 2 Newe Ya’ar Research Center, Ramat, Yishay 30095 Israel; 3 United States Department of Agriculture, Agricultural Research Service, and Department of Horticulture, University of Wisconsin, 53706 Madison, Wisconsin USA

**Keywords:** Plant sciences, Secondary metabolism

## Abstract

Carrot (*Daucus carota* L.) is an important root vegetable crop with high nutritional value, characteristic flavor, and benefits to human health. *D. carota* tissues produce an essential oil that is rich in volatile terpenes and plays a major role in carrot aroma and flavor. Although terpene composition represents a critical quality attribute of carrots, little is known about the biosynthesis of terpenes in this crop. Here, we functionally characterized 19 terpene synthase (TPS) genes in an orange carrot (genotype DH1) and compared tissue-specific expression profiles and *in vitro* products of their recombinant proteins with volatile terpene profiles from DH1 and four other colored carrot genotypes. In addition to the previously reported (*E*)-β-caryophyllene synthase (*Dc*TPS01), we biochemically characterized several TPS proteins with direct correlations to major compounds of carrot flavor and aroma including germacrene D (*Dc*TPS7/11), γ-terpinene (*Dc*TPS30) and α-terpinolene (*Dc*TPS03). Random forest analysis of volatiles from colored carrot cultivars identified nine terpenes that were clearly distinct among the cultivars and likely contribute to differences in sensory quality. Correlation of TPS gene expression and terpene metabolite profiles supported the function of *Dc*TPS01 and *Dc*TPS03 in these cultivars. Our findings provide a roadmap for future breeding efforts to enhance carrot flavor and aroma.

## Introduction

Carrot (*Daucus carota* L.) is one of the most nutritionally and economically valuable root crops worldwide. As a member of the Apiaceae family, carrot was first domesticated in the form of yellow and purple root varieties more than 1000 years ago, followed by breeding of orange varieties around the 16^th^ century in Europe^1,2^. Carrot breeding has focused largely on enhancing the content of alpha- and beta-carotene as precursors of vitamin A and improving root morphology and disease or pest resistance^3,4^. In addition, increased attention has been placed on developing carrot cultivars with different aroma and flavor attributes^5^.

Carrot produces an essential oil that directly contributes to its aroma and flavor. The oil consists predominantly of blends of volatile 10-carbon monoterpenes and 15-carbon sesquiterpenes that reside in highly interconnected phloem oil ducts in the above- and belowground tissues^5,6^. Specific sensory attributes have been associated with different terpenes^7^. For example, accumulation of monoterpenes often leads to a harsh and bitter flavor or a burning aftertaste, which reduces overall palatability^5,6^. The mixtures of terpenes together with non-volatile phenolics and sugars are highly genotype specific and affect the sensory qualities of carrot genotypes of different color^5–8^. Orange cultivars exhibit high intensities of “carrot” flavor and aroma in contrast to yellow cultivars^5^. Purple cultivars are known to have a considerably sweeter flavor^5^, while red genotypes have been associated with a higher intensity of green “carrot top” aromas and bitter flavors based on lower levels of sugars and higher concentrations of specific terpene compounds (e.g. β-pinene)^5^. A general increase in harsh taste occurs when carrots experience environmental stress such as elevated temperature conditions (>18 °C)^8,9^. This change is directly correlated with an increase in terpene levels presumably masking the perception of sweet taste^9^. To facilitate breeding of carrots with desirable sensory qualities and maintain these qualities under stress conditions, a better understanding of the genetic determinants of carrot aroma and flavor in general, and terpenes in particular, is required.

Terpenes are biosynthesized from the 5-carbon isoprenoid precursor isopentenyl diphosphate (IDP) and its isomer dimethylallyl diphosphate (DMADP), which are derived from the plastidial methylerythritol phosphate (MEP) pathway or the mevalonic acid (MVA) pathway in the cytosol/ER and peroxisomes^10^. Condensation reactions between IDP and DMADP lead to the formation of *cis*- or *trans*-prenyl diphosphates that include geranyl diphosphate (GDP, C_10_), neryl diphosphate (NDP, C_10_), (*Z*,*Z*)-farnesyl diphosphate (FDP, C_15_), and geranylgeranyl diphosphate (GGDP, C_20_) in plastids, and (*E*,*E*)-FDP in the cytosol. The prenyl diphosphates are then further converted in these compartments by enzymes of the terpene synthase (TPS) family into structurally diverse volatile monoterpenes and sesquiterpenes or semi-volatile and non-volatile diterpenes (C_20_). The TPS superfamily is divided into seven sub-families^11^ with TPSs from angiosperms residing in families a, b, c, e/f, and g. TPS-a and TPS-b subfamilies include primarily sesqui-TPSs and mono-TPSs, respectively, while di-TPSs are found in the c and e/f clades. Mono-TPSs, sesqui-TPSs, and di-TPSs representing the g subfamily typically make linear terpenes and lack the highly conserved RRX_8_W motif characteristic of mono-TPSs of the TPS-b clade. TPS genes often undergo species specific divergence and duplications resulting in terpene metabolic plasticity and adaptations^12^. While the structural diversity and biosynthetic evolution of terpenes have been studied extensively in a variety of crops (e.g. maize, tomato, strawberry, peppermint)^13–16^, only two TPS genes from carrot, *DcTPS01* and *DcTPS02*^17^, have been functionally analyzed to date, leaving a majority of the biosynthetic genes responsible for the biosynthesis of carrot terpene volatiles uncharacterized. Recently, the genome of the orange, doubled-haploid, Nantes-type carrot DH1 has been sequenced^18^. Genomic and transcriptomic analyses of this genotype estimated a family of 36 potentially functional TPS genes^18^. However, the latest analysis of the carrot TPS gene family predicted 65 full-length TPSs^19^. In conjunction with this study, several QTLs associated with TPS genes were predicted to correlate with distinct terpene compounds. To investigate these loci in more detail and determine the major enzymes contributing to carrot aroma and flavor, we performed biochemical characterizations of 19 carrot TPS genes based on their expression profiles in different tissues of DH1 (leaves, petioles and roots) and roots of field-grown colored carrot varieties (Red, Orange, Yellow and Purple). Employing random forest analysis, we determined distinct terpene representatives of each cultivar and predicted the TPS genes responsible for their biosynthesis based on cultivar-specific transcriptome profiles. As terpene content strongly affects carrot flavor and aroma^20^, results from this study can be applied to enhance carrot palatability and overall carrot quality.

## Experimental Results

### Analysis of terpene volatiles in DH1 carrot leaves, petioles and roots

**Figure 1:**
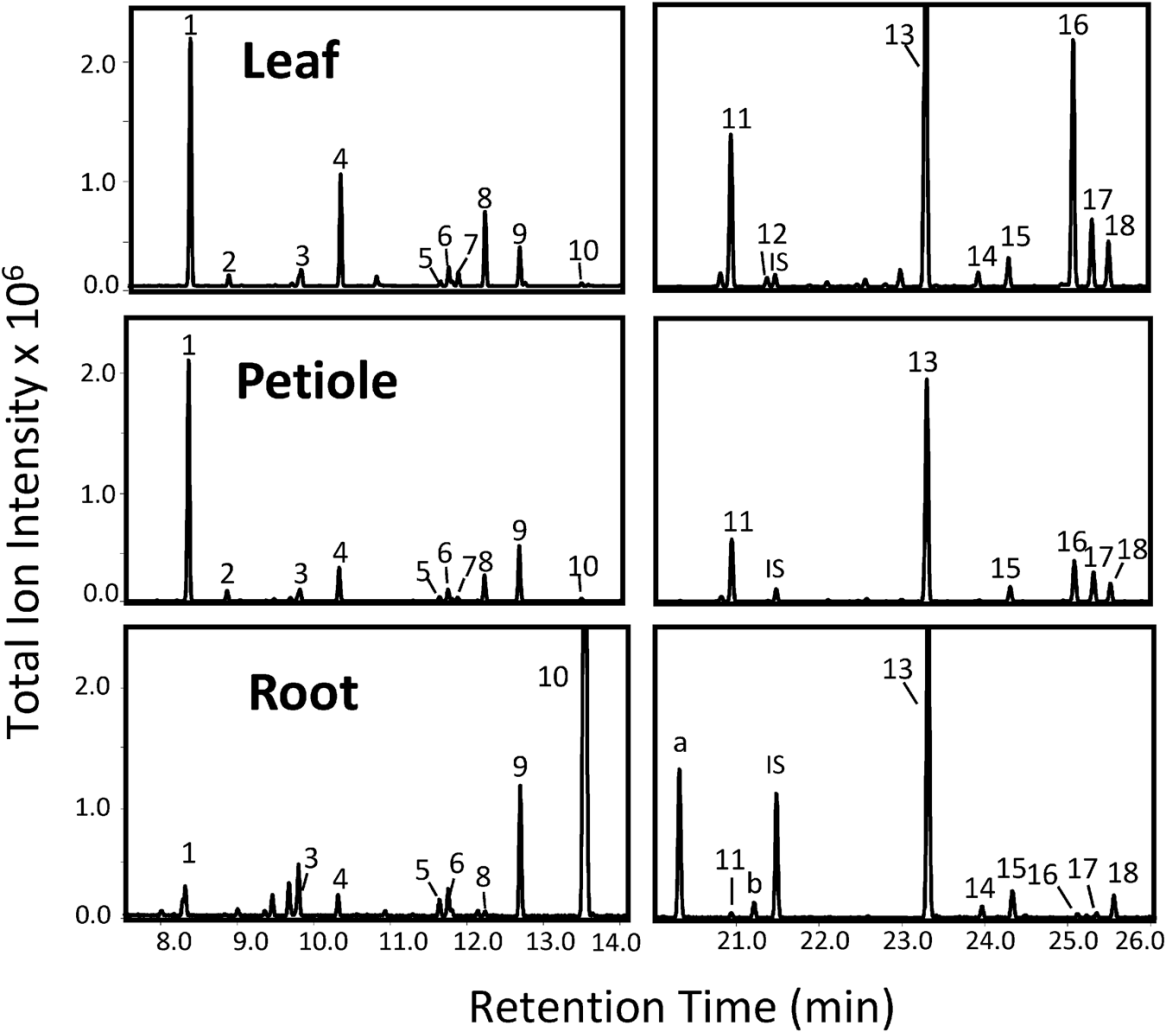
GC-MS analysis of hexane extracts from 11-week old carrot (DH1) leaves, petioles and roots. Left and right panels show compounds of the same chromatogram. The root extract was injected with a four times lower split ratio than the leaf and petiole extracts. 1: α-pinene*, 2: camphene*, 3: β-pinene*, 4: β-myrcene*, 5: cymene, 6: limonene*, 7: (*Z*)-β-ocimene*, 8: (*E*)-β-ocimene*, 9: γ-terpinene*, 10: α-terpinolene*, 11: δ-elemene, 12: longipinene, 13: (*E*)-β-caryophyllene*, 14: (*E*)-β-farnesene*, 15: α-humulene*, 16: germacrene D*, 17: α-farnesene, 18: β-bisabolene*, IS: internal standard 1-bromodecane. *indicates compounds that were identified with authentic standards or by comparison with compounds of Opopanax oil. Other compounds were identified by library comparison only. a and b, unidentified compounds with similarity confidence values <90%.

Volatile terpenes were extracted from leaves, petioles, and roots of the doubled-haploid carrot DH1 and qualitatively and quantitatively analyzed using GC-MS and GC-FID, respectively. We found that the tissues contained a diverse blend of terpene compounds including 18 major monoterpenes and sesquiterpenes (Fig. 1). Leaf tissues contained high levels of the monoterpenes α-pinene, β-myrcene and (*E*)-β-ocimene, and the sesquiterpenes δ-elemene, (*E*)-β-caryophyllene and germacrene D (Fig. 1; Supplementary Table S1). Comparable profiles were obtained from petioles with the exception of lower levels of β-myrcene and germacrene D (Fig. 1; Supplementary Table S1). Root tissues showed reduced levels of α-pinene, and increased levels of γ-terpinene and α-terpinolene compared to above ground tissues (Fig. 1; Supplementary Table S1). Other putative sesquiterpene volatiles were not reported due to low levels of abundance and lack of authentic standards or oils for verification.

### Identification of TPS Gene Models in the Carrot Genome

The carrot reference genome (Phytozome *v12*, *Daucus carota v2.0*, DH1), and publically available RNA-seq data sets (SRA SAMN03216637, cv. DH1) were queried for TPS genes using NCBI TBLASTX. We identified 52 putative TPS gene models including the 36 TPS genes previously predicted from DH1 by Iorizzo, *et al*.^18^. Although Iorizzo, *et al*.^18^ previously generated a TPS nomenclature based on chromosomal positioning, we adopted the most recent TPS naming system for *D. carota* proposed by Keilwagen, *et al*.^19^. Comparisons of the 52 TPS gene models against the reference genome revealed 43 unique full-length open reading frames (Table 1). Several TPS genes are located in biochemical gene clusters on chromosomes 1, 3, 4, 5, 7 and 8, including a dense five gene cluster on chromosome 4 (Table 1, Supplementary Fig. S1). Additional TPS gene models predicted by Keilwagen, *et al*.^19^ in a genome-wide association study (GWAS) were not pursued further due to low transcript levels in roots, inability to amplify a full-length transcript, or identity with previously annotated TPSs (Supplementary Figs. S2 and S3).

**Table 1:** Characteristics of the 43 terpene synthase genes analyzed in this study organized by genomic location. ^a^Unique transcripts identified by Keilwagen, *et al*.^19^, and ^b^previously characterized by Yahyaa, *et al*.^17^.

TPS	Locus ID	Genomic Location	Genomic Cluster	No. of Exons	cDNA Constructed	TPS Sub-family
*Dc*TPS01	*DC*AR_023152	Chr6:1181665..1185241	None	7	Yes^b^	a
*Dc*TPS32	*DC*AR_002080	Chr1:24861393..2486203	None	7	No	b
*Dc*TPS45	*DC*AR_002829	Chr1:33280604..33282540	1	7	No	g
*Dc*TPS46	*DC*AR_002830	Chr1:33286015..33288129		7	No	g
*Dc*TPS19	*DC*AR_002831	Chr1:33293414..33302528		7	Yes	g
*Dc*TPS47	*DC*AR_004091	Chr1:44627888..44628091	None	7	No	b
*Dc*TPS25	*DC*AR_012483	Chr3:47468861..47475243	None	15	Yes	c
*Dc*TPS52	*DC*AR_012537	Chr3:48081099..48082521	2	7	No	b
*Dc*TPS30	*DC*AR_012538	Chr3:48088855..48092222		7	Yes	b
*Dc*TPS09	*DC*AR_012965	Chr4:33893835..33896155	3	7	No	b
*Dc*TPS02	*DC*AR_012963	Chr4:33914246..33916610		7	Yes^b^	b
*Dc*TPS26	DCAR_013310	Chr4:31144998..31147390	4	8	Yes	b
*Dc*TPS04	DCAR_013298	Chr4:31217904..31220266		7	Yes	b
*Dc*TPS54	DCAR_013297	Chr4:31227164..31230361		7	Yes	b
*Dc*TPS55	DCAR_013294	Chr4:31244459..31247374		7	Yes	b
*Dc*TPS27	DCAR_013293	Chr4:31249549..31251992		7	Yes	b
*Dc*TPS56	*DC*AR_016843	Chr5:8253832..8257662	5	14	No	e
*Dc*TPS28	*DC*AR_016844	Chr5:8267895..8275147		13	Yes	e
*Dc*TPS14	*DC*AR_017536	Chr5:20668670..20671917	None	7	Yes	b
*Dc*TPS17	*DC*AR_018214	Chr5:27521963..27529973	None	7	No	b
*Dc*TPS57	*DC*AR_018422	Chr5:29664251..29668971	None	14	No	c
*Dc*TPS33	*DC*AR_019208	Chr5:37087498..37094271	None	7	No	b
*Dc*TPS59	*DC*AR_019490	Chr5:39497726..39502226	None	15	No	c
*Dc*TPS23	*DC*AR_024752	Chr7:18911173..18913238	6	7	Yes	g
*Dc*TPS60	*DC*AR_024753	Chr7:18917227..18919574		7	No	g
*Dc*TPS43	*DC*AR_026971	Chr8:27108437..27111829	7	7	No	b
*Dc*TPS44	*DC*AR_026972	Chr8:27097599..27100665		7	No	b
*Dc*TPS29	*DC*AR_027915	Chr8:17430080..17434674	None	12	No	f
*Dc*TPS62	*DC*AR_028138	Chr8:14626722..14629317	None	7	No	b
*Dc*TPS16	*DC*AR_032119	S3773:14141..17230	None	7	No	b
*Dc*TPS15	None^a^	Chr3:2698521..2703290	None	7	Yes	a
*Dc*TPS38	None^a^	Chr4:15499493..15500247	None	7	No	a
*Dc*TPS42	None^a^	Chr2:1678067..1678357	None	7	Yes	a
*Dc*TPS10	None^a^	Chr1:44680386..44685155	None	7	Yes	b
*Dc*TPS11	None^a^	Chr1:28341531..28346300	None	7	Yes	a
*Dc*TPS05	None^a^	Chr3:45432441..45440095	None	7	No	b
*Dc*TPS03	None^a^	Chr2:39586545..39589031	None	7	Yes	b
*Dc*TPS07	None^a^	Chr9:8999311..9003484	None	7	Yes	a
*Dc*TPS53	None^a^	Chr3:48692713..48694881	None	7	Yes	a
*Dc*TPS12	None^a^	Chr3:45451840..45455295	None	7	No	b
*Dc*TPS48	None^a^	Chr1:44677421..44686660	None	7	Yes	b
*Dc*TPS13	None^a^	Chr4:25547281..25566360	None	7	No	a
*Dc*TPS21	None^a^	Chr1:45337229..45352534	None	7	No	b

**Figure 2:**
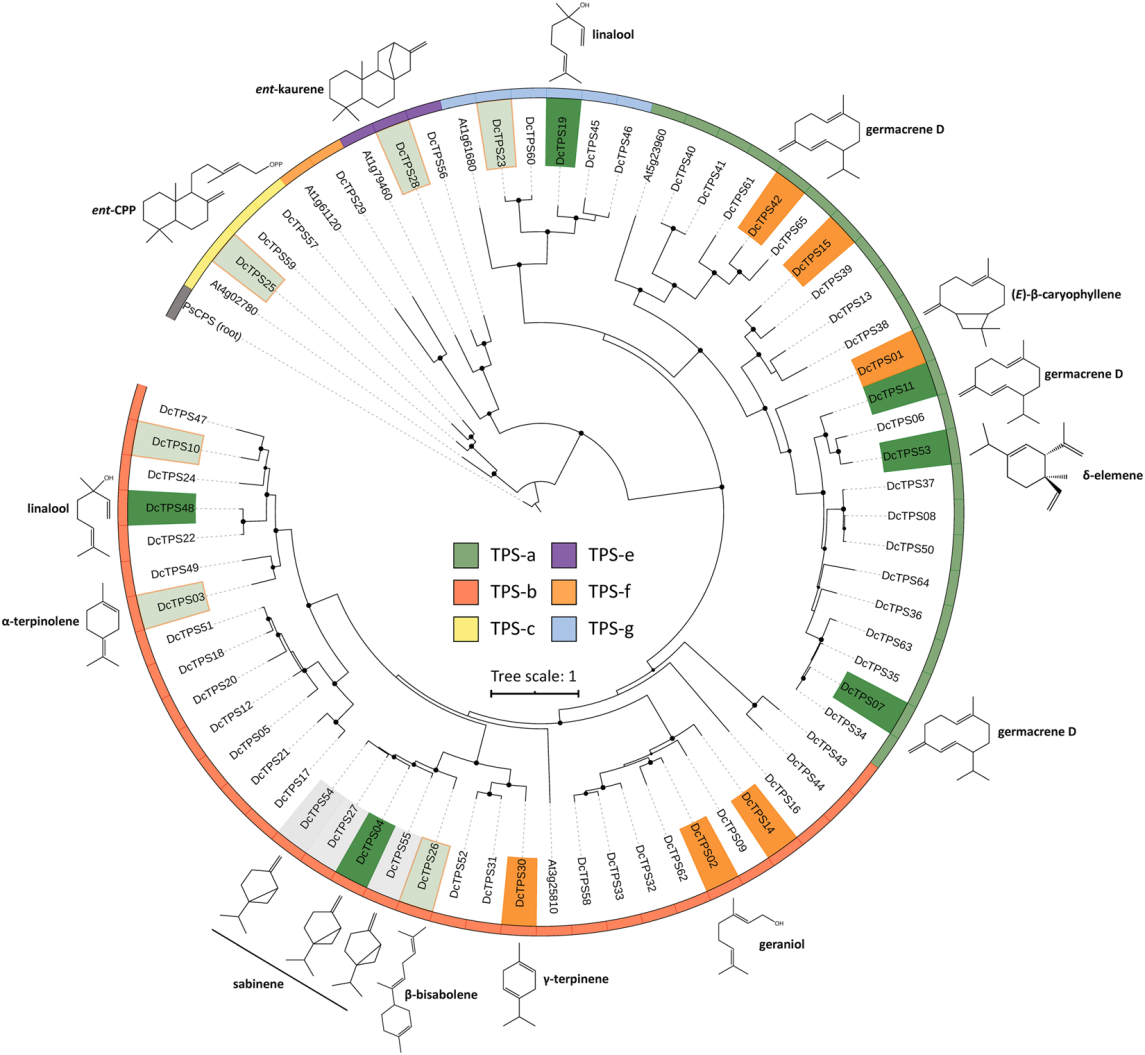
Maximum-likelihood phylogenetic tree of predicted and functionally characterized TPSs in *Daucus carota* (DH1) and select TPSs from Arabidopsis. Numbering of TPSs is according to Keilwagen *et al*.^19^ Circles indicate bootstrap support of >80% where bootstrap replicates = 500. The tree was rooted with the gymnosperm *ent*-CPP synthase from *Picea sitchensis* (*Ps*CPS). Clades representing different TPS subfamilies are indicated by different colors in the perimeter of the tree. TPSs that were functionally characterized in this study are marked with colored boxes and their primary enzymatic products are shown on the outside of the perimeter. TPSs marked in orange are primarily expressed in root tissue while TPSs marked in green are predominantly expressed in the petiole or leaves. TPSs expressed in above- and belowground tissues are marked in light green boxes with an orange shape outline. Functionally characterized TPSs without tissue-specific expression data are marked in grey.

Amino acid alignment and phylogenetic analysis of the 43 TPS proteins indicated that carrot TPSs are organized in six TPS sub-families according to the classification by Chen, *et al*.^11^ (Fig. 2; Supplementary Figs. S4, S5, S6, S7, S8, and S9). We found that eight members cluster in the TPS-a sub-family (*Dc*TPS01, *Dc*TPS07, *Dc*TPS11, *Dc*TPS13, *Dc*TPS15, *Dc*TPS38, *Dc*TPS42 and *Dc*TPS53) including the previously characterized (*E*)-$\beta$-caryophyllene synthase *Dc*TPS01^17^. ChloroP analysis of subcellular localization indicated no putative transit peptides across the TPS-a clade, suggesting putative activity as sesqui-TPSs converting (*E,E*)*-*FDP in the cytosol (Supplementary Table S2). The TPS-b clade spans 22 members, of which 12 were predicted to carry plastidial transit peptide sequences (*Dc*TPS02*, Dc*TPS03*, Dc*TPS04, *Dc*TPS09*, Dc*TPS10, *Dc*TPS27*, Dc*TPS30, *Dc*TPS33, *Dc*TPS48*, Dc*TPS52*, Dc*TPS54*, Dc*TPS55) suggesting these proteins are targeted to plastids where they convert GDP into monoterpenes (Fig. 2; Supplementary Table S2). We identified five type-g TPSs (*Dc*TPS19, *Dc*TPS23, *Dc*TPS45, *Dc*TPS46 and *DcT*PS60), of which only *Dc*TPS19 was predicted to function as a mono-TPS based on a putative plastidic transit peptide (Fig. 2 and Supplementary Table S2). The three members of the TPS-c clade (*Dc*TPS25*, Dc*TPS57, and *Dc*TPS59) were predicted to encode class II diterpene synthases based on the presence of the conserved DxDD motif required for the protonation-initiated cyclization of GGDP into bicyclic prenyl diphosphates including copalyl diphosphate^21^. The TPS-e/f subfamily contains 3 members (*Dc*TPS28*, Dc*TPS29, and *Dc*TPS56) and generally includes predicted class I di-TPSs and mono-/sesqui-TPSs.

### Gene candidate selection

**Figure 3:**
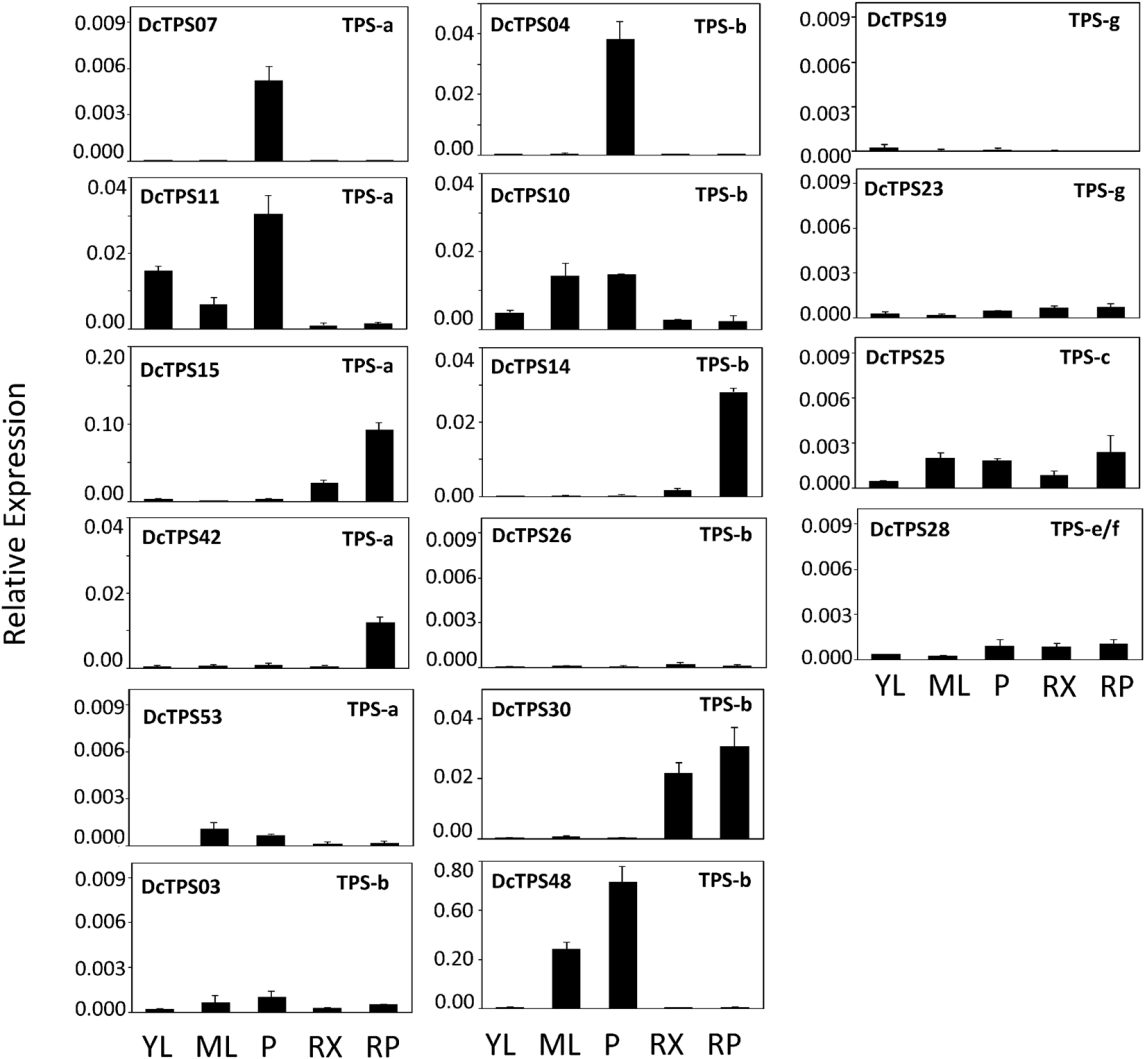
qRT-PCR analysis of transcript abundance of TPS genes that were functionally characterized (except *DcTPS*54 and *DcTPS*55). Relative expression levels across tissues for each gene were calculated using the ΔΔC_T_ standard method normalized to expression of actin. Amplifications were performed in biological and technical triplicate and error bars indicate standard deviation from the mean. YL: young leaf, ML: mature leaf, P: petiole, RX: root xylem and RP: root phloem.

Gene candidates for biochemical characterization were first screened by tissue specific RNA-seq analysis of DH1 and root specific RNA-seq analysis of colored carrots (Supplementary Figs. S2 and S3). TPS gene candidates with high in silico transcript levels were further selected based on the ability to obtain full-length transcripts and real time qRT-PCR amplicons across multiple tissues (Fig. 3; Supplementary Fig. S10). Full-length cDNAs or cDNAs with truncated plastidial transit peptides (19 in total) were constructed for all root-expressed TPS genes (*DcTPS03*, *DcTPS10*, *DcTPS11*, *DcTPS14*, *DcTPS15*, *DcTPS25*, *DcTPS26*, *DcTPS28* and *DcTPS30*), genes with high expression in above ground tissues (*DcTPS04*, *DcTPS07*, *DcTPS19*, *DcTPS23*, *DcTPS42*, *DcTPS48*, *DcTPS53*) and any additional TPS genes associated with QTLs (*DcTPS27*, *DcTPS54* and *DcTPS55*) identified by Keilwagen, *et al*.^19^. *In vitro* TPS assays with the recombinant partially purified TPS proteins were performed using common TPS substrates (GDP, NDP, (*E*,*E*)-FDP, (*Z*,*Z*)-FDP and GGDP) and terpene products were analyzed by headspace SPME-GC-MS.

### Characterization of TPS-a Clade Genes

**Figure 4:**
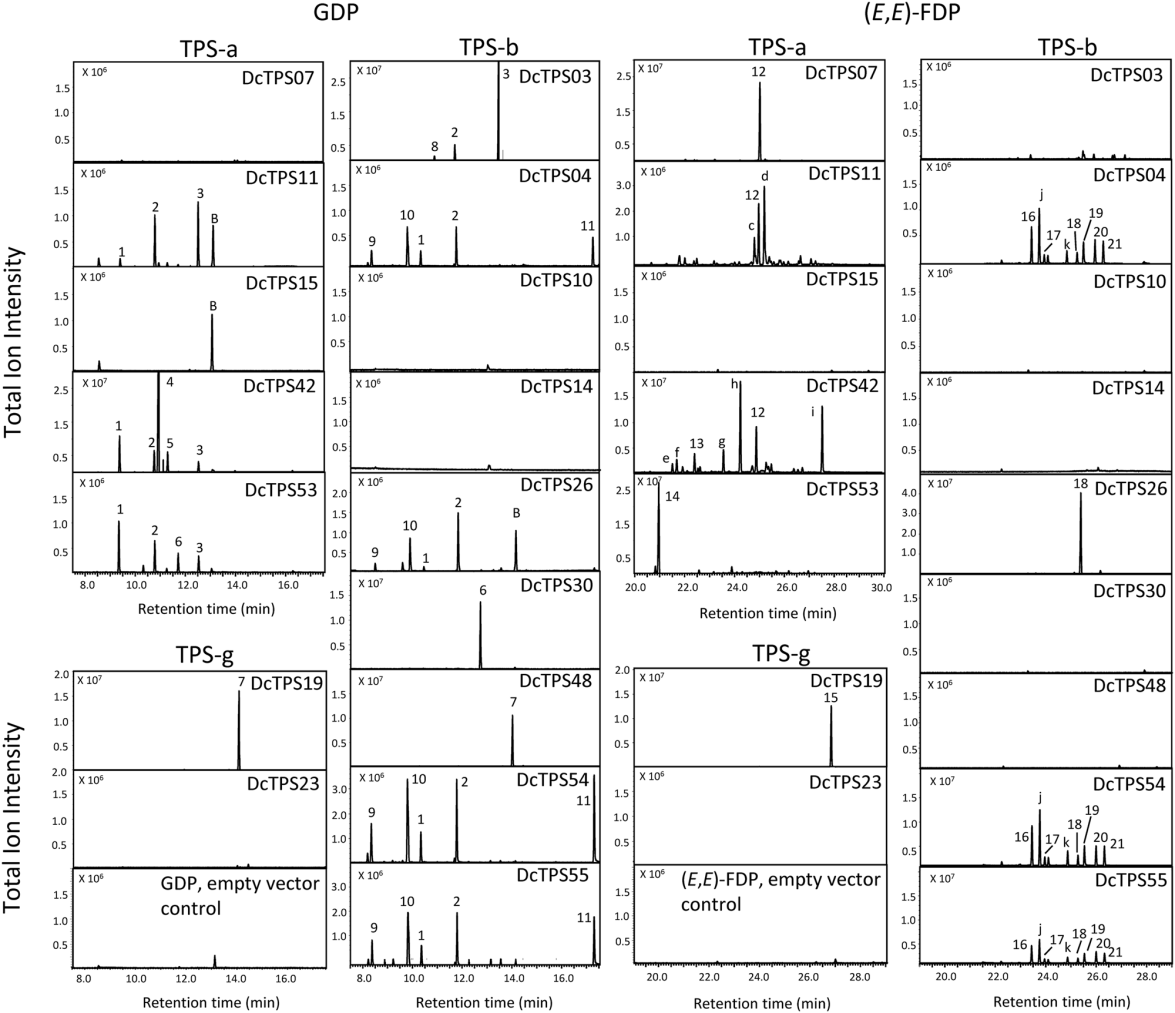
SPME-GC-MS analysis of terpene products from assays with recombinant TPS enzymes. Partially purified proteins were incubated in the presence of GDP or (*E*,*E*)-FDP. 1: β-myrcene*, 2: limonene*, 3: α-terpinolene*, 4: (*Z*)-β-ocimene*, 5: (*E*)-β-ocimene*, 6: γ-terpinene*, 7: linalool*, 8: α-phellandrene*, 9: α-pinene*, 10: sabinene*, 11: α-terpineol*, 12: germacrene D*, 13: β-elemene, 14: δ-elemene, 15: nerolidol*, 16: (*E*)-α-bergamotene*, 17: (*E*)-β-farnesene*, 18: (*Z*)-α-bisabolene*, 19: β-bisabolene*, 20: sesquiphellandrene*, 21: (*E*)-α-bisabolene*. *indicates compounds that were identified with authentic standards or by comparison with compounds of Opopanax oil. Other compounds were identified by library comparison only (≥95% confidence level). Mass spectra of these compounds are shown in Supplementary Figure S12. Lower case letters indicate additional terpene compounds with lower identification confidence levels. Mass spectra of these compounds are depicted in Supplementary Figure S13. Note that retention times of products obtained from TPS-a enzymes with GDP are shifted by 1 min due to difference in column condition. B; SPME fiber-related background occurring in some assays.

In addition to *DcTPS01*, which was previously reported as an (*E*)-$\beta$-caryophyllene synthase^17^, five full-length cDNAs were isolated for TPS-a type genes *DcTPS07, DcTPS11, DcTPS15, DcTPS42*, and *DcTPS53* based on expression profiling as described above. *DcTPS11* was found to be most highly expressed in aboveground tissues including young leaves, matures leaves and petioles (Fig. 3). The recombinant DcTPS11 protein converted (*E,E*)-FDP into germacrene D as one of its major enzymatic products (Fig. 4). Similarly, *DcTPS07*, which showed highest transcript abundance in petioles (Fig. 3), encodes a protein that exclusively formed germacrene D from (*E,E*)-FDP (Fig. 4). As germacrene D is a major component of the carrot essential oil in aboveground tissues, it is likely that both *Dc*TPS11 and *Dc*TPS07 contribute to the formation of this compound *in vivo*. Another member of the TPS-a subfamily, *DcTPS53*, was expressed in mature leaves and the petiole and its recombinant protein was found to convert (*E,E*)*-*FDP to δ-elemene as a major product and constituent of DH1 leaf terpenes (Figs. 3 and 4). The recombinant protein of the root-expressed gene *DcTPS15* had limited activity with all tested substrates (Fig. 4; Supplementary Fig. S11). Enzyme assays with recombinant *Dc*TPS42 demonstrated that the enzyme produced several putative sesquiterpene products from (*E,E*)-FDP including germacrene D (Fig. 4). Additional members of the TPS-a clade were not tested based on previous characterization (*DcTPS01*^17^), low levels of constitutive expression, or inability to amplify a full-length transcript (*DcTPS13* and *DcTPS38*).

We also examined all characterized TPS-a type proteins for their ability to accept GDP and GGDP as well as the *cis*-prenyl diphosphates NDP and (*Z*,*Z*)-FDP as substrates. *Dc*TPS11 catalyzed the formation of monoterpenes (limonene, α-terpinolene) from GDP and made a γ-bisabolene isomer from (*Z,Z*)-FDP (Fig. 4; Supplementary Fig. S11). Interestingly, *Dc*TPS11 did also convert GGDP into a cembrene-like diterpene (Supplementary Fig. S14). *Dc*TPS53 converted (*Z*,*Z*)-FDP into bisabolenes and another putative sesquiterpene, and accepted GDP and NDP to make β-myrcene, limonene, γ-terpinene, and α-terpinolene (Fig. 4; Supplementary Fig. S11). *Dc*TPS42 converted GDP, NDP and (*Z*,*Z*)-FDP to the monoterpene products β-myrcene and β-ocimene, limonene and α-terpinolene, and an α-bisabolene isomer, respectively (Fig. 4; Supplementary Fig. S11). Several terpenes produced by the TPS-a type proteins from these alternative substrates are components of the DH1 terpene blends (Fig. 1). However, it remains unclear whether these enzymatic reactions occur *in vivo* given the predicted cytosolic localization of the TPS-a enzymes and presumed limited availability of GDP, NDP, (*Z*,*Z*)-FDP, and GGDP in this compartment.

### Characterization of TPS-b Clade Genes

Of the 22 genes in the TPS-b subfamily, *Dc*TPS02 was previously identified as a monoterpene synthase converting GDP into β-myrcene and geraniol^17^. We further functionally characterized ten TPS b-type proteins (*Dc*TPS03, *Dc*TPS04, *Dc*TPS10, *Dc*TPS14, *Dc*TPS26, *Dc*TPS27, *Dc*TPS30, *Dc*TPS48, *Dc*TPS54 and *Dc*TPS55), of which all except *Dc*TPS14 and *Dc*TPS26 carry putative plastidial transit peptides (Supplementary Table S2).

*DcTPS03*, predicted to encode a root expressed mono-TPS based on transcriptome analysis, was found to be expressed at low levels in all tested tissues (Fig. 3). The truncated recombinant *Dc*TPS03 protein converted GDP into α-terpinolene, which is a dominant component of carrot root essential oil (Fig. 1). In addition, *Dc*TPS03 produced the monoterpenes α-phellandrene and limonene from GDP (and NDP) (Fig. 4; Supplementary Fig. S11).

Five genes in the TPS-b clade (*DcTPS04*, *DcTPS26*, *DcTPS27*, *DcTPS54* and *DcTPS55*) were previously reported to reside in a dense TPS gene cluster on chromosome 4 and correlate with a QTL for sabinene and terpinen-4-ol production in roots (Table 1, Supplementary Fig. S1)^19^. *Dc*TPS04 and *Dc*TPS26 share ~88% sequence identity with a major difference attributed to the presence of a putative 44 amino acid plastidial transit peptide in *Dc*TPS04 (Supplementary Fig. S5). Truncated *Dc*TPS04 and full-length *Dc*TPS26 produced similar volatile profiles with sabinene, limonene, β-myrcene, α-pinene, and α-terpineol from GDP (and NDP) (Fig. 4; Supplementary Fig. S11). The same compounds were made by recombinant *Dc*TPS54 and *Dc*TPS55 from GDP (and NDP) (Fig. 4; Supplementary Fig. S11). A full-length cDNA was obtained for *Dc*TPS27; however, the presence of an unspliced ~1 kb intron downstream of the first exon introduced a premature stop codon and the gene was therefore not further tested. It is possible that the plastid-targeted *Dc*TPS04, *Dc*TPS54 and *Dc*TPS55 proteins synthesize sabinene in roots although we did not detect this monoterpene as a major compound in DH1 tissues and found *DcTPS04* to be most highly expressed in the petiole (Figs. 1 and 3).

*In vitro* enzyme assays with a truncated *Dc*TPS30 protein led to the conversion of GDP (and NDP) into $\gamma$-terpinene as the major product (Fig. 4; Supplementary Fig. S11). Because of the predominant expression of the *DcTPS30* gene in DH1 roots it is likely that this gene is responsible for the accumulation of high levels of $\gamma$-terpinene in this tissue (Figs. 1 and 3). Expression of the gene *DcTPS48* was only detected in aboveground tissues and transcripts were highly enriched in mature leaves and petioles (Fig. 3). The partially purified *Dc*TPS48 enzyme converted GDP (and NDP) into linalool, which could only be found at low levels in mature leaves (Fig. 4; Supplementary Fig. S11). The recombinant proteins of *DcTPS10* and *DcTPS14*, although expressed in above and/or root tissues, did show only limited or no activity with any tested substrates (Figs. 3 and 4; Supplementary Fig. S11). Other members of the TPS-b clade were not tested based on previous characterization (*Dc*TPS02)^17^, low levels of constitutive expression, or inability to amplify a full-length transcript (Supplementary Fig. S2; *Dc*TPS05, *Dc*TPS09, *Dc*TPS12, *Dc*TPS16, *Dc*TPS17, *Dc*TPS21, *Dc*TPS32, *Dc*TPS33, *Dc*TPS47, *Dc*TPS52, and *Dc*TPS62).

Several of the characterized recombinant TPS-b type proteins also converted C_15_ and C_20_ prenyl diphosphate substrates under *in vitro* conditions; however, the contribution of these reactions to sesquiterpene and diterpene formation in planta remains unclear based on the plastidial localization of the proteins, limited substrate availability, or absence of the enzymatic product in planta. Recombinant *Dc*TPS03 and *Dc*TPS48 showed limited sesquiterpene production with (*E,E*)*-*FDP but made several bisabolene isomers from (*Z,Z*)-FDP (Supplementary Fig. S11). *Dc*TPS04 and *Dc*TPS26 produced several sesquiterpenes from (*E,E*)*-*FDP (and (*Z,Z*)-FDP) including α-bergamotenes (*Dc*TPS04) and β-bisabolene (*Dc*TPS26) (Fig. 4; Supplementary Fig. S11). In addition, *Dc*TPS26 did convert GGDP into an unidentified diterpene hydrocarbon product (Supplementary Fig. S14).

### *Dc*TPS19 and *Dc*TPS23 are Members of the TPS-g Subfamily

Based on sequence similarity to characterized genes in the TPS-g subfamily^22^, and the presence of a putative plastidial transit peptide, we predicted the recombinant protein of gene *Dc*TPS19 to function as a mono-TPS (Supplementary Fig. S6). *DcTPS19* was found to be expressed at low levels in all tested tissues except young leaves (Fig. 3). The *Dc*TPS19 protein converted GDP (and NDP) into linalool but also accepted (*E,E*)-FDP (and (*Z*,*Z*)-FDP) as substrates to make nerolidol (Fig. 4; Supplementary Fig. S11). Linalool could only be detected at low levels in leaves and may be further modified *in vivo* to non-volatile derivatives, e.g. by glycosylation. Another gene in the TPS-g family, *DcTPS23*, showed low expression in all tissues with highest transcript levels in petioles and roots (Fig. 3). Enzymatic activity of the recombinant *Dc*TPS23 protein was limited with all substrates (Fig. 4; Supplementary Fig. S11). The remaining genes in the TPS-g subfamily (*DcTPS45*, *DcTPS46*, and *DcTPS60*) were not characterized based on low levels of expression in roots or inability to amplify full-length cDNAs.

### *Dc*TPS25 Belongs to the TPS-c Clade

**Figure 5:**
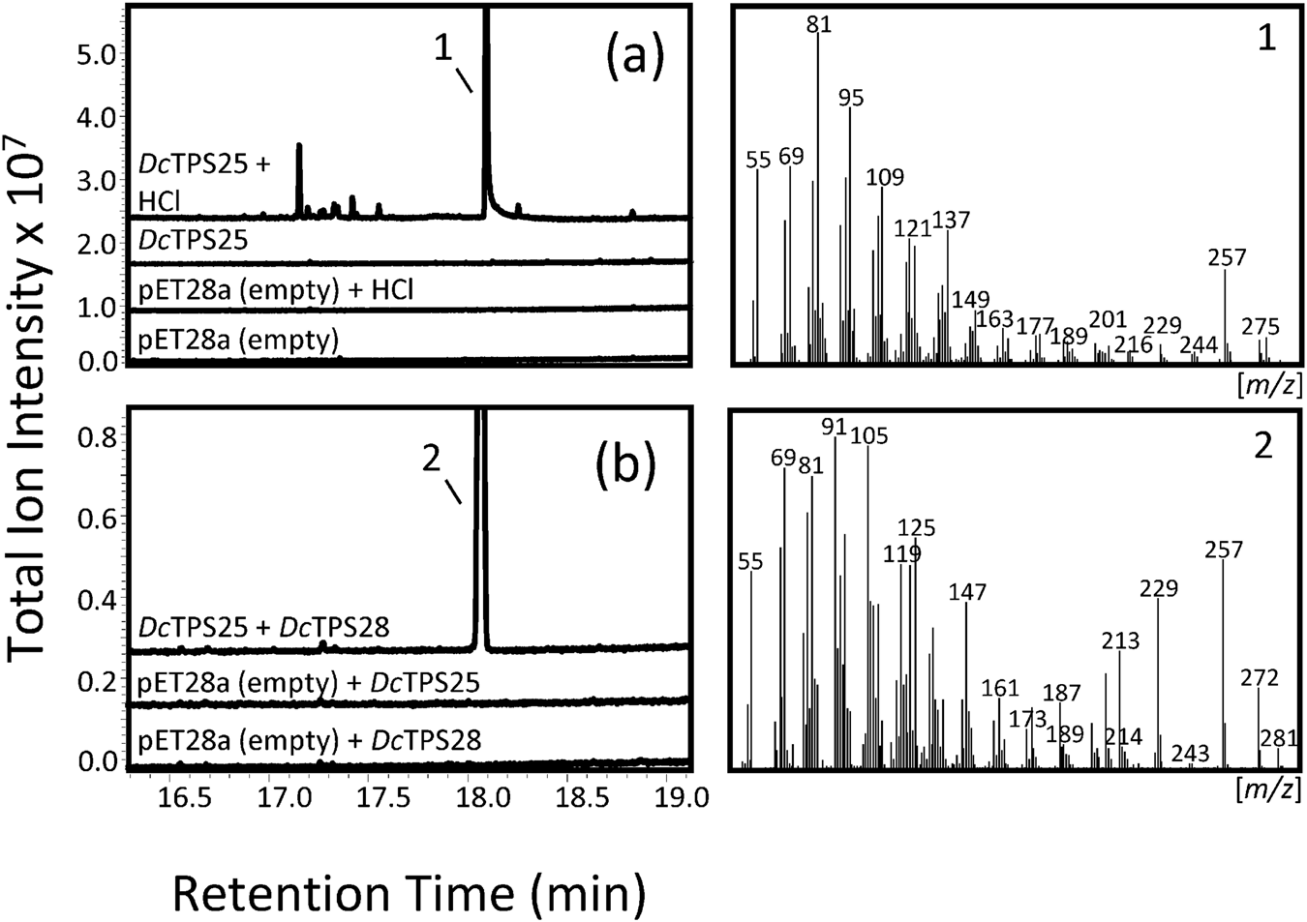
GC-MS analysis of terpene products from an assay with *Dc*TPS25 enzyme **(a)** and a combined assay with *Dc*TPS25 and *Dc*TPS28 **(b)** enzymes. Partially purified, recombinant proteins were incubated in the presence of GGDP. Enzyme products are shown in the upper chromatograms. Chromatograms from empty vector controls are presented below. 1: *ent*-copalol (dephosphorylated *ent*-CPP); 2: *ent*-kaurene. Compound identification is based on library comparisons (NIST/WILEY) and comparisons to enzymatic products from known *ent-*CPP and *ent*-kaurene synthases. Mass spectra of 1 and 2 are presented in the right panel.

The plant TPS-c subfamily comprises enzymes with an N-terminal $\gamma$-domain characteristic of diterpene synthases involved in primary and secondary metabolism. In carrot, we identified three TPS genes in the TPS-c subfamily, of which *DcTPS25* was expressed in above and belowground tissues in contrast to low expression of genes *DcTPS57* and *DcTPS59* (Fig. 3; Supplementary Fig. S10). The recombinant *Dc*TPS25 protein was found to function as a class II diterpene cyclase converting GGDP into *ent*-copalyl diphosphate (CDP) based on mass spectral comparison of the acid hydrolyzed product *ent*-copalol (Fig. 5a) with *ent*-copalol derived from the *Arabidopsis thaliana* copalyl diphosphate synthase. No enzymatic activity was detected with any other substrate tested.

### *Dc*TPS28 in an *ent-*Kaurene Synthase in the TPS-e/f Subfamily

Of the three TPS-e/f type genes identified by RNA-seq analysis (*DcTPS28, DcTPS29* and *DcTPS56*), we focused on *DcTPS28* based on its expression in roots (Fig. 3). When the recombinant *Dc*TPS28 was tested for class I diterpene synthase activity with GGDP as substrate, no product was detected. However, when co-expressed with a pGGeC plasmid carrying a *GGDPS* gene from *Abies* grandis and a *CPS* gene from *Arabidopsis thaliana*^23^, *Dc*TPS28 converted *ent*-CDP into *ent*-kaurene (Fig. 5b). *ent-*Kaurene could also be produced by co-incubating partially purified *Dc*TPS25 and *Dc*TPS28 with GGDP confirming the enzymatic activities of both enzymes (Fig. 5b). Production of *ent-*kaurene was verified by mass spectral comparison to products from a known *ent*-kaurene synthase of *Bradyrhizobium japonicum*.

### Diverse colored root cultivars exhibit distinct volatile terpene profiles

**Figure 6:**
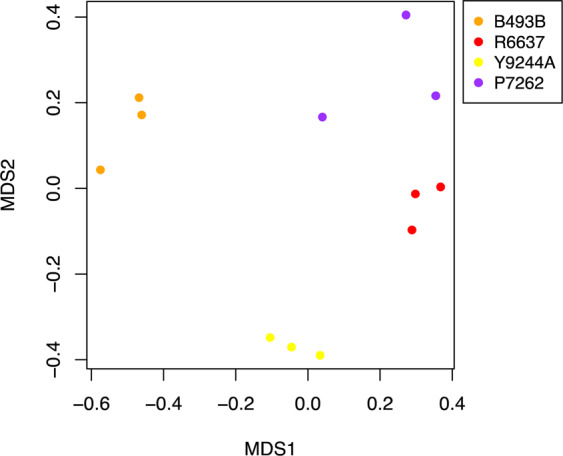
Multidimensional scaling plot (MDS) based on a random forest analysis of root volatile terpenes from field grown colored carrots. Yellow-Y9244A, Orange-B493B, Red-R6637 and Purple-Orange-P7262.

**Figure 7:**
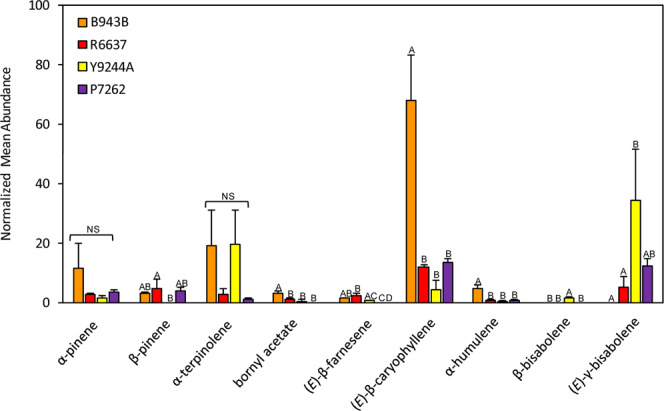
Comparison of relative terpene levels for compounds identified by random forest analysis. Letters indicate significant differences determined by ANOVAs and post-hoc Tukey-HSD comparisons (conducted separately for each compound following a significant MANOVA). Yellow-Y9244A, Orange-B493B, Red-R6637 and Purple-Orange-P7262. NS, not significant. Error bars indicate standard deviation across three biological replicates (n = 3). Statistically significant differences were considered when p ≤ 0.05 with α = 0.05.

Carrot cultivars of different color can be distinguished by distinct sensory qualities. To determine whether these differences correlate with modifications in terpene profiles, we performed a random forest analysis (see Methods for details) of 14 major monoterpene and sesquiterpene compounds in the colored cultivars P7262 (purple), R6637 (red), Y9244A (yellow) and B493B (orange) (Supplementary Fig. S15). This analysis revealed a strong separation of the colored genotypes (Fig. 6). Variable selection, using the R package Boruta, identified nine terpene factors as important in distinguishing the colored varieties (Table S5). We found that orange carrot roots in this study (cv. B493B) accumulated significantly higher levels of (*E*)-β-caryophyllene (ANOVA; p = 2.95e-05), α-humulene (ANOVA; p = 1.03e-04) and bornyl acetate (ANOVA; p = 4.23e-04) compared to red, purple and yellow cultivars (Fig. 7). In addition, yellow carrots (cv. Y9244A), accumulated high levels of β-bisabolene (ANOVA; p = 2.02e-03) and (*E*)-γ-bisabolene (ANOVA; p = 7.51e-03) in comparison to the other tested cultivars (Fig. 7). Although α-terpinolene significantly contributed to cultivar differences (Table S5; ANOVA; p = 0.046), no significant pairwise differences were detected among cultivars (Fig. 7). To determine if the observed cultivar specific terpene differences correlated with the expression of particular TPS genes, we analyzed TPS transcript levels from RNA-seq data of all cultivars using the Bioconducter package Limma (Fig. S2). We found that the cultivar-specific transcript profile of *DcTPS01* with highest levels in the orange cultivar overlapped with the metabolite profile of (*E*)-β-caryophyllene and α-humulene supporting the function of *DcTPS01* as an (*E*)-β-caryophyllene in planta. In addition, increased α-terpinolene levels in yellow and orange carrots correlated with the transcript profiles of the α-terpinolene synthase *DcTPS03*. Several TPS genes exhibited highest transcript levels in the yellow cultivar (Fig. S2). Of these genes, *DcTPS26* may contribute to the formation of β-bisabolene in yellow rooted carrots since the DcTPS26 protein lacks a plastidial transit peptide and might make β-bisabolene from (*E*,*E*)-FDP in the cytosol (Fig. 4). Three other genes (*DcTPS03*, *DcTPS04, DcTPS54*) may have similar roles since their corresponding enzymes are targeted to plastids, where they may contribute to synthesizing γ-bisabolenes and β-bisabolene from (*Z*,*Z*)-FDP (Fig. S11). Proteins encoded by other TPS genes with highest expression in the yellow cultivar either did not make bisabolenes or have not been functionally characterized (*Dc*TPS10, *Dc*TPS16, *Dc*TPS33, *Dc*TPS42). No additional correlations between TPS genes expression and profiles of other terpenes were found due to multiple enzymes being involved in the formation of several terpenes (e.g. α-pinene, β-pinene, β-farnesene) or unknown biochemical origin of the compound (bornyl acetate).

## Discussion

Carrot (*Daucus carota* L.) has been extensively studied for its commercial and nutritional value, essential oil content, and resistance against pathogens and herbivores^24,25^. Volatile terpene constituents of carrot essential oil were first analyzed 50 years ago^26^, but their genetic determinants have largely remained unidentified. Here we report on the major terpene volatiles of the orange, doubled-haploid carrot DH1, whose genome was recently sequenced^18^, and identify several TPS enzymes involved with the formation of these compounds in the DH1 and other colored carrot genotypes.

Despite the substantial variation of terpene composition in different carrot genotypes, several of the highly abundant terpenes detected in leaves, petioles, and roots of the DH1 genotype occur also at high levels in other cultivars^19^. These compounds include the monoterpenes α-pinene and β-myrcene and the sesquiterpenes (*E*)-β-caryophyllene and germacrene D in leaves and α-terpinolene and (*E*)-β-caryophyllene in roots. DH1 leaves and roots also contain high amounts of δ-elemene and γ-terpinene, respectively, which have been identified at various levels in other cultivars^19,27^. By contrast, bornyl acetate, a typical terpene extracted from carrot roots, was only observed in trace amounts in DH1 root tissue. Compound profiles in the petiole were similar to those in leaves but proportionally fewer terpenes were detected in this tissue. Except for (*E*)-β-caryophyllene, which is the most predominant volatile in both leaves and roots, DH1 above and belowground tissues maintain distinct terpene profiles^19^. These tissue specific blends differ largely at a quantitative rather than qualitative scale, which suggests possible movement of compounds throughout the plant. As interconnected phloem oil ducts occur in carrot roots, petioles and leaves^6^, it is conceivable that terpenes are mobilized to some extent from roots to shoots or vice versa through schizogenous spaces. The presence of oil ducts in the phloem would suggest that terpene compounds reside mostly in this tissue; however, we did not observe major differences in terpene content between root phloem and xylem under our preparation conditions.

Our initial search of TPS gene models in the DH1 reference genome and publicly available transcriptomes, yielded 43 unique full-length genes. The TPS genes reside on all chromosomes and frequently occur in gene clusters indicating multiple gene duplication events^19^ (Table 1, Supplementary Fig. S1). Genes encoding putative cytochromes P450 are associated with some of these clusters (Supplementary Fig. S1) suggesting possible oxidations of terpene olefins although major immediate oxidation products are typically not detected in extracts of carrot tissues. Notably, the type-b clade in the carrot TPS gene family has undergone a substantial expansion in comparison to TPS families of other dicots^28^ (Fig. 2) suggesting a selection for monoterpene biosynthetic genes in domesticated carrot. By contrast, the carrot TPS genome contains few di-TPS genes in the TPS-c and e/f clades, two of which (*Dc*TPS25, *Dc*TPS28) could be associated with the formation of CPP and kaurene required for gibberellin biosynthesis. These genes were among 19 out of the 43 genes, which we selected for biochemical characterization based on transcript abundance and the ability to obtain full length cDNAs. qRT-PCR and RNA-seq derived transcript profiles were generally in agreement for root-expressed TPS genes but showed more tissue-specific variation in aboveground tissues. Recently, Keilwagen, *et al*.^19^ identified 22 additional full length TPS genes in the DH1 genome, most of which we did not pursue because of their low transcript levels in roots or inability to amplify full-length transcripts.

The enzymatic products of many of the characterized mono-TPS and sesqui-TPS proteins are present in leaf or root tissues indicating that these enzymes contribute to the detected terpene mixtures depending on their expression profiles and subcellular localization. Several of the recombinant proteins did also convert the *cis*-isoprenyl diphosphates NDP and (*Z*,*Z*)-FDP *in vitro*. It is unclear whether these diphosphate intermediates are synthesized in carrot tissues and serve as enzymatic substrates in planta. However, a search of the DH1 genome for isoprenyl diphosphate synthases identified two genes that cluster with *cis*-isoprenyl diphosphate synthases from *Solanum lycopersicum* and, therefore, may encode enzymes with similar activity (Supplementary Fig. S16).

Besides the previously characterized (*E*)-β-caryophyllene synthase DcTPS01, we identified TPS enzymes that are most likely responsible or contribute to the formation of five predominant monoterpenes and sesquiterpenes in leaves and roots of DH1 and presumably other genotypes: *Dc*TPS03 produces mostly α-terpinolene and *Dc*TPS30 makes $\gamma$-terpinene as its major product. Both γ-terpinene and α-terpinolene have been associated with a sweet, fruity, and citrus like odor^7,29,30^ and add to a terpene flavor and burning aftertaste^5^. A correlation of *Dc*TPS03 with the formation of α-terpinolene was also supported from analysis of colored cultivars (see below). However, since *DcTPS03* was expressed at fairly low levels in DH1 based on our qRT-PCR results, another uncharacterized root-expressed TPS might contribute to the formation of α-terpinolene in this genotype.

Keilwagen, *et al*.^19^ identified a QTL on chromosome 4 for the monoterpene sabinene and its conversion product terpinen-4-ol, which they associated with a cluster of five closely related genes in the TPS-b clade. Sabinene has been characterized as a compound involved with carrot top aroma^7^. We indeed found four of the genes on this QTL (*DcTPS04*, *DcTPS26*, *DcTPS54* and *DcTPS55*), to encode proteins that catalyze the formation of sabinene among other monoterpenes. Three of them are likely to exhibit this activity *in vivo* because of their targeting to plastids. Other QTLs predicted for γ-terpinene (*Dc*TPS29) and bornyl acetate (*Dc*TPS03) could not be confirmed either because of low transcript abundance (*Dc*TPS29) or different catalytic activity (*Dc*TPS03 makes mostly α-terpinolene) suggesting further refinement of QTL associations will be required. *Dc*TPS04, *Dc*TPS26, *Dc*TPS54 and *Dc*TPS55 may also contribute to the synthesis of α-pinene and β-myrcene in above- and belowground tissues. α-pinene and β-myrcene have been described with a pinene, carrot top odor and a green, terpene like odor, respectively^7^. Among the sesquiterpene synthases *Dc*TPS07, *Dc*TPS11 and *Dc*TPS42 were found to produce germacrene D and *Dc*TPS53 catalyzes the formation of δ-elemene; both compounds are major constituents of leaf volatile terpenes.

We further tested whether terpenes distinctive of selected colored cultivars could be associated with particular TPS genes. Random forest analysis identified several terpene factors with significant differences in four colored cultivars, which likely contribute to the variation in sensory attributes (Figs. 6 and 7). Correlation of TPS gene expression and terpene metabolite profiles of the cultivars supported the function of TPS01 as (*E*)-β-caryophyllene synthase with highest expression in the orange cultivar, TPS03 as α-terpinolene synthase mostly active in the orange and yellow genotypes, and possible roles of *Dc*TPS26, *Dc*TPS04*, Dc*TPS54, and *Dc*TPS03 in contributing to β-bisabolene and γ-bisabolene formation, respectively, in yellow carrots (Fig. 7, Supplementary Fig. S2).

Taken together, we have identified genes in the large carrot TPS family that are likely responsible for the formation of predominant terpene compounds in above and belowground tissues including several aroma and flavor constituents in roots. Results from this study may be directly applied in future breeding efforts to improve the sensory quality of carrots.

## Material and Methods

### Plant growth and conditions

Seeds from the doubled haploid orange Nantes type carrot DH1 were kindly provided by Rijk Zwaan and directly seeded into 6-inch clay pots filled with 50% potting mix and 50% composted soil. Seedlings were grown at the University of Wisconsin, Madison, Walnut Street Greenhouse under a 12-h photoperiod with an average temperature cycle of 20–25 °C (night/day). Colored carrot cultivars (yellow-Y9244A, orange-B493B, red-R6637 and purple-7262) were field grown at the University of Wisconsin, Madison. Whole plants were harvested 100 days after planting and frozen immediately in liquid nitrogen for later isolation of RNA and metabolite extraction. Three individual plants from each cultivar were used for extraction.

### Identification of TPS genes in the carrot genome

Publically available RNA-seq data for above- and belowground tissues were retrieved from the NCBI Short Read Archive^18^, biosample SAMN03216637, and quality assessed using FastQC. Reads were truncated by nine bp using Trimmomatic^31^ to remove low quality sequences and assembled de novo using Trinity^32^. Assembled transcriptomes (20 total) were individually queried with a representative TPS sequence (*Dc*TPS01) using TBLASTX. The resulting “hits” were manually curated for putative functionality based on length and presence of aspartate rich conserved motifs (DDxxD, DxDD). Gene models were refined further by comparing transcripts to genome sequences available in Phytozome (*Daucus carota v2.0*). Exon/intron structure was predicted by alignment of coding sequences to genomic sequences using the Gene Structure Display Server^33^. Putative N-terminal plastidial transit peptides were predicted from multiple sequence alignments and by analysis of each sequence using the transit peptide prediction software ChloroP^34^. Phylogenetic analysis was conducted in Geneious (v8.0.2) using default settings (bootstrap = 1000) based on multiple sequence alignments generated with Clustal Omega^35^.

### TPS Gene expression analysis in DH1 tissues

#### Initial RT-PCR Analysis of 43 Carrot TPS Genes

Total RNA was extracted from each DH1 tissue type in biological triplicate (young leaves, fully expanded leaves, petiole, root xylem, root phloem and whole root) using the TRIzol Plus RNA Purification Kit (Life Technologies, Carlsbad, CA) in accordance with the manufacturer’s protocol. RNA was treated for DNA contamination with the TurboDNA-free kit (Life Technologies, Carlsbad, CA) and used for first strand cDNA synthesis with SuperScriptII reverse transcriptase and oligo(dT)_18_ primers (Invitrogen) according to the manufacturer’s instructions. PCR amplification of 43 TPS genes was performed with each cDNA, gene specific primers (Supplementary Table S3), and Taq DNA Polymerase (Promega) with an initial denaturing step of 95 °C for 5 min, followed by denaturation for 30 s at 95 °C, annealing for 30 s at 50 °C, extension for 1 min at 72 °C and a final extension for 7 min for 30 cycles. Actin and PP2A were used as internal controls.

#### qRT-PCR analysis of transcript abundance of 43 TPS Genes

Total RNA extraction and first-strand cDNA synthesis were performed as described above, however RNA was first normalized between samples and replicates to 2.5 µg based on denaturing gel electrophoresis and spectrophotometer measurements at 260 nm. The resulting cDNA was diluted to 100 ng/µl. Reactions were performed with 1 µl cDNA in a 20 µl reaction using Power SYBR Green PCR master mix (Applied Biosystems) and gene specific primers (Supplementary Table S3). PCR amplifications were done with a CFX96 Touch real-time PCR detection system (Bio-Rad) with the following cycles: 95 °C for 10 min, followed by 40 cycles of 95 °C for 15 s, 50 °C 30 s and 60 °C for 1 min. Melt curve analysis was performed at the end of amplification to ensure specificity of each primer pair. Relative expression levels across tissues for each TPS gene were calculated using the relative quantification method and normalized to actin^36^.

### RNA-seq Analysis of TPS Gene Expression in Colored Carrots

Total RNA was extracted from 14 week old whole roots, of colored carrot cultivars (B493B, R6637, Y9244A, P7262), with three roots (i.e. three biological replicates) per sample set. Total RNA was extracted using the TRIzol Plus RNA Purification Kit (Life Technologies, Carlsbad, CA) following the manufacturer’s protocol. DNA was removed with the ‘DNA free-kit’ provided with the RNA purification kit. RNA quantification was measured on a Nanodrop One Spectrophotometer and quality control was done on an Agilent 2100 Bioanalyzer RNA NanoChip. For each RNA sample, libraries were prepared at the University of Wisconsin-Madison Gene Expression Center and sequenced on an Illumina HiSeq 2000 using 1×100 nt reads. After quality control with FastQC (http://www.bioinformatics.babraham.ac.uk/projects/fastqc/), reads were filtered with Trimmomatic version 0.32 with adapter trimming and using a sliding window of length ≥50 and Phred quality score ≥28^31^. Reads were mapped against the carrot genome sequence (GenBank accession LNRQ01000000.1) using Bowtie2 (Langmead and Salzberg 2012)^37^. Illumina reads were mapped against the carrot genome sequence (GenBank accession LNRQ01000000.1) using Rsubread version 1.24.2^38^. Transcript expression was analyzed using the Bioconductor package limma^39^.

### Amplification of Full-Length TPS cDNAs and Plasmid Construction

Full-length cDNAs for *DcTPS07*, *DcTPS11*, *DcTPS15*, *DcTPS19*, *DcTPS23*, *DcTPS26*, *DcTPS42*, *DcTPS53* and those truncated based on predicted transit peptide coding regions *DcTPS03*, *DcTPS04*, *DcTPS10*, *DcTPS14*, *DcTPS25*, *DcTPS27*, *DcTPS28*, *DcTPS30*, *DcTPS48*, *DcTPS54* and *DcTPS55* were obtained by PCR-amplification with gene-specific primers carrying restriction sites (Supplementary Table S4). Template cDNA was derived from root and stem RNA as described above. Amplification was performed with Q5 High-Fidelity DNA polymerase in a 25 µl reaction volume with the following PCR conditions: 98 °C for 30 s, followed by 30 cycles of 98 °C for 30 s, 55 °C for 30 s, 72 °C for 1 min 45 s and a final extension at 72 °C for 2 min. Amplified fragments were gel purified using a NucleoSpin Gel and PCR clean-up kit (Macherey-Nagel, MN) and concentrated to ~10 µl. A 10 µl A-tailing reaction was prepared with 3 µl of purified PCR product incubated in the presence of 10 mM dATPs and Taq polymerase at 72 °C for 30 min. The resulting product was ligated overnight into the pGEM-T Easy vector (Promega) and Sanger sequenced to verify the insert. Open reading frames were then digested with the appropriate restriction enzymes (typically *Bam*HI*, Xho*I) and ligated overnight into the corresponding restriction sites of the bacterial expression vector pET28a (Novagen). Constructs were Sanger sequenced again prior to expression in *Escherichia coli*.

### Recombinant protein expression in *E. coli* and TPS assays

Plasmids were transformed into *E. coli* BL21-CodonPLus(DE3) cells (Stratagene), and individual colonies were selected for inoculation into 5 ml Luria-Bertani (LB) media supplemented with 50 µM kanamycin and grown at 37 °C/220 rpm overnight. The following day, 1 ml of the saturated overnight culture was transferred to 100 ml LB media supplemented with 50 µM kanamycin and grown at 37 °C/220 rpm until the optical density reached 0.5–0.7. After cooling cultures at ~25 °C for 30 min, 0.5 mM isopropyl 1-thio-ß-D-galactopyranoside (IPTG) was added to induce protein production and cultures were incubated at 18 °C/220 rpm for 16 h. Cell pellets were washed with 10 mM Tris base and 50 mM potassium chloride, resuspended in 4 ml phosphate buffered saline (PBS, 50 mM sodium phosphate, 100 mM sodium chloride, 10% glycerol) supplemented with 1 mM dithiothreitol (DTT) and 0.5 mM phenylmethylsulfonyl fluoride (PMSF), and lysed by sonication. Clarified extracts were mixed with equal parts PBS and recombinant His(6×)-tagged proteins were partially purified by Ni^2+^ affinity chromatography according to the manufacturer’s instructions (Qiagen). Partially purified proteins were then desalted on PD-10 desalting columns (GE) equilibrated with assay buffer (10 mM MOPSO, 10% glycerol [v/v] and 1 mM DTT, pH 7.0) and visualized by SDS-PAGE (10%, GenScript). *In vitro* enzyme assays were prepared by combing Ni-NTA purified protein with 20 mM MgCl_2_ and 60 µM commercially available prenyl diphosphate substrates (Echelon Biosciences) in a 125 µl reaction volume in a 10 ml screw cap vial (Supelco). Vials were immediately sealed and incubated for 5 min at 30 °C in the presence of a 100-µM polydimethylsiloxane fiber (Supelco) using automated solid phase microextraction (SPME, AOC-5000 Shimadzu). Incubation was extended to 40 min in assays with *Dc*TPS04, *Dc*TPS53, *Dc*TPS54, and *Dc*TPS55 proteins. Volatile compounds were eluted by thermal desorption for 4 min at 240 °C and separated and analyzed by gas chromatography mass spectrometry (GC-MS-QP2010S, Shimadzu). Eluted compounds were separated on a Zebron capillary column (30 m x 0.25 mm i.d. x 0.25 µm, Phenomenex) in a 5:1 split using Helium as the carrier gas (1.4 ml min^−1^ flow rate) and a temperature gradient increasing from 40 °C (2 min initial hold following injection) to 220 °C at a rate of 5 °C min^−1^. Identification of major volatile compounds was confirmed by comparisons of retention times and mass spectra to authentic standards when available (Sigma), mass spectral libraries (Wiley and NIST) and Opopanax essential oil (Floracopeia).

### *Dc*TPS25 Assay

Diterpene cyclase activity of *Dc*TPS25 was tested by incubating partially purified protein as described above with 60 µM GGDP and 10 mM MgCl_2_ for 1 h at 30 °C with the addition of a 1 ml hexane overlay. Following incubation, 80 µl of 5 M HCl or water was added and mixed by vortex to facilitate acid hydrolysis of terpene products. Hexane fractions were dried over magnesium sulfate (MgSO_4_), concentrated to ~40 µl, and 1 µl was injected into the GC-MS as described above. Identification of *ent*-copalol was confirmed by mass spectral comparisons to acid hydrolysis products from the known CPS from *Arabidopsis thaliana*^23^.

### *Dc*TPS28 Assay

Diterpene synthase activity of TPS28 was tested as described above, either alone or co-incubated with partially purified TPS25, and by co-expression of pET28a-*Dc*TPS28 with a pGGeC plasmid (provided by Dr. Reuben Peters), which carries a *GGDPS* gene from *Abies* grandis and a *CPS* gene from *Arabidopsis thaliana*^23^. Constructs, including a known *ent*-kaurene synthase gene from *Bradyrhizobium japonicum* as a control (pDEST15-BjKS courtesy of Dr. Reuben Peters), were co-transformed into *E. coli* C41 (DE3) OverExpress cells (Lucigen) and a single bacterial colony was selected to inoculate 5 ml LB media and incubated for 16 h at 37 °C. The saturated culture was used to inoculate a 50 ml TB culture, which was incubated at 37 °C until the OD_600_ reached 0.5–0.7. Protein expression was induced by the addition of 0.5 mM IPTG and incubated with shaking at 18 °C for 72 h. Cultures were extracted with equal parts hexane, dried over MgSO_4_, concentrated to ~40 µl, and 1 µl was injected for GC-MS analysis as described above. Identification of *ent*-kaurene was achieved as described above, and by comparisons to the *ent*-kaurene product produced by the BjKS.

### GC-MS and GC-FID analysis of terpenes from plant tissues

Volatile terpenes were extracted from 1 g of leaf, petiole, root phloem, root xylem and whole root samples each from three individual plants (DH1) grown under culture conditions described above. Samples were rinsed with deionized water, dried with tissue paper and immediately frozen in liquid nitrogen for processing. Samples were then ground to a fine powder for 2 min in the presence of liquid nitrogen, weighed, transferred to 5 ml hexanes and mixed by vortex for 20 s. The ground material was placed in an ultrasonic bath (Fisher Scientific) for 10 min and then pelleted by centrifugation. Following the collection of two fractions, 1-bromodecane was added for a final concentration of 20 ng/µl as an internal standard and extracts were dried over a MgSO_4_ column and concentrated on ice to ~40 µl. Extracts were separated as above with a 10:1 or 40:1 split using the same column and conditions above, and by GC-FID (Thermo Finnigan) using Helium as the carrier gas (1.4 ml min^−1^ flow rate) and Nitrogen, Hydrogen and Air (25, 35, 350 ml min^−1^, respectively) as makeup and combustion gasses. Annotation of major terpene compounds was achieved as described above. Chromatograms were compared between GC-MS and GC-FID results for compound identification and quantification using the multipoint internal standard method (Alltech). Standard curves for monoterpene and sesquiterpene compounds were constructed with authentic α-pinene and α-humulene (Sigma), respectively, and obtained values were normalized to gram fresh weight. Analysis of volatile compounds for colored carrot cultivars (B493B, R6637, Y9244A, P7262) followed identical methodology with the exception that compounds were only analyzed in roots by GC-MS and reported as normalized relative abundance ((peak area analyte/peak area internal standard)/gram fresh weight).

### Random forest analysis and boruta factor selection

To assess the importance of major terpene compounds from roots in distinguishing among colored carrot cultivars (see above), relative terpene abundances from each sample (n = 3 per cultivar) were analyzed by random forest classification models, followed by variable selection using the Boruta algorithm in R v3.5.0^40,41^. Random forest is a machine-learning classification method that builds sets of decision trees from bootstrapped subsets of the entire sample set. Each tree classifies a subset of samples according to a random sample of their attributes (in this case different VOCs) and then calculates the classification error according to the remaining unselected samples. By averaging across thousands of iterative trees, this method provides a robust estimation of which compounds are most important in distinguishing among groups^42^. Random forest analysis was set to 5000 bootstrap iterations for compound selection. To further assess the direction and significance of compound differences across cultivars, compounds selected in bootstrapped models were retained for use in a MANOVA model conducted across all selected compounds. Based on significant multivariate differences among cultivars (F = 16.936, p = 6.6e-5), this was followed with ANOVAs and Tukey-HSD post-hoc comparisons of compound levels among cultivars.

## Supplementary information

Supplementary information.

## Data Availability

RNA-seq data sets used for TPS gene identification and expression in *D. carota* DH1 are available in the NCBI Short Read Archive (SRA)^18^, biosample SAMN03216637. RNA-seq data sets for the colored carrot cultivars are available in the SRA database under BioProject accession number PRJNA594937.
