# Publisher Correction: Diversity and function of terpene synthases in the production of carrot aroma and flavor compounds

**DOI:** 10.1038/s41598-020-76732-9

**Published:** 2020-11-10

**Authors:** Andrew Muchlinski, Mwafaq Ibdah, Shelby Ellison, Mossab Yahyaa, Bhagwat Nawade, Suzanne Laliberte, Douglas Senalik, Philipp Simon, Susan R. Whitehead, Dorothea Tholl

**Affiliations:** 1 Department of Biological Sciences, Virginia Tech, Blacksburg, VA 24061 USA; 2 Newe Ya’ar Research Center, 30095 Ramat, Yishay, Israel; 3 United States Department of Agriculture, Agricultural Research Service, and Department of Horticulture, University of Wisconsin, Madison, WI 53706 USA

Correction to: *Scientific Reports*, 10.1038/s41598-020-66866-1, published online 19 June 2020

A reference cited as reference 43 in the body of the Supplementary Information is omitted from the References section in this Article.

The paper is listed below as Reference 43.

Larkin, M. A. *et al.* Clustal W and Clustal X version 2.0. *Bioinformatics* **23**, 2947–2948 (2007).

