# Supplementary material for: Diversity and function of terpene synthases in the production of carrot aroma and flavor compounds
Source: Sci Rep. 2020 Jun 19;10:9989. doi: 10.1038/s41598-020-66866-1 (PMC7305226; doi:10.1038/s41598-020-66866-1)
Supplement: Supplementary file 1 — Supplementary information. [file 41598_2020_66866_MOESM1_ESM.pdf]

Supplementary Information

Diversity and function of terpene synthases in the production of carrot aroma and flavor compounds

Andrew Muchlinski[1], Mwafaq Ibdah[2], Shelby Ellison[3], Mossab Yahyaa[2], Bhagwat Nawade[2], Suzanne Laliberte[1], Douglas Senalik[3], Philipp Simon[3], Susan R. Whitehead[1], and Dorothea Tholl[1]

[1]Department of Biological Sciences, Virginia Tech, Blacksburg, Virginia, USA 24061 (A.M., S. L., S.W., D.T.)

[2]Newe Ya'ar Research Center (M.Y., B.N., M.I.), Ramat Yishay 30095, Israel

[3]United States Department of Agriculture, Agricultural Research Service, Department of Horticulture, University of Wisconsin, Madison, Wisconsin, USA 53706 (S.E., D.S., P.S.)

SUPPLEMENTARY TABLES

Supplementary Table S1. Volatile terpenes from carrot tissues (n=3, three individual plants) quantified by GC-FID using multi point internal standard calibration curves of authentic α-pinene and α-humulene. Identification was based on comparison to mass spectral libraries (NIST, Wiley) and comparison (*) to authentic standards or compounds in an Opopanax essential oil. Values are reported as micrograms per gram fresh weight.

Compound	Leaf	Petiole	Root	Root Phloem	Root Xylem
α-pinene*	6.00 ± 0.39	3.71 ± 0.12	1.19 ± 0.01	1.01 ± 0.01	1.06 ± 0.00
camphene*	0.26 ± 0.07	0.17 ± 0.09	trace	trace	trace
β-pinene*	0.59 ± 0.00	0.30 ± 0.00	0.21 ± 0.02	0.07 ± 0.00	0.07 ± 0.02
β-myrcene*	3.54 ± 0.44	0.40 ± 0.35	0.07 ± 0.07	0.08 ± 0.04	0.10 ± 0.04
p-cymene	0.06 ± 0.20	0.05 ± 0.02	0.05 ± 0.03	trace	trace
limonene*	0.46 ± 0.00	0.29 ± 0.05	0.09 ± 0.00	0.05 ± 0.02	0.08 ± 0.03
(*E*)-β-ocimene*	1.87 ± 0.4	0.43 ± 0.1	trace	trace	trace
α-terpinolene*	0.1 ± 0.02	0.06 ± 0.03	0.85 ± 0.10	0.03 ± 0.00	0.02 ± 0.02
γ-terpinene*	0.88 ± 0.30	0.76 ± 0.05	0.41 ± 0.20	0.39 ± 0.09	0.39 ± 0.20
(*E*)-β-caryophyllene*	12.73 ± 0.10	5.61 ± 0.20	2.13 ± 1.17	1.22 ± 0.40	1.70 ± 1.40
δ-elemene	4.57 ± 0.60	1.41 ± 0.06	0.05 ± 0.00	0.07 ± 0.02	0.20 ± 0.01
α-humulene*	0.92 ± 0.02	0.36 ± 0.03	0.12 ± 0.08	0.12 ± 0.06	0.17 ± 0.13
germacrene-D*	4.26 ± 0.93	0.68 ± 0.15	0.02 ± 0.00	0.03 ± 0.01	0.04 ± 0.03
β-bisabolene*	1.34 ± 0.02	0.45 ± 0.06	0.08 ± 0.00	0.13 ± 0.03	0.35 ± 0.02

Supplementary Table S2. Predicted transit peptides based on ChloroP (v1.1) analysis of predicted full-length *Dc*TPS proteins.

Name	Length	cTP	cTP-length (amino acids)
*Dc*TPS01	560	-	-
*Dc*TPS02	594	Y	36
*Dc*TPS03	589	Y	40
*Dc*TPS04	608	Y	43
*Dc*TPS05	550	-	-
*Dc*TPS07	563	-	-
*Dc*TPS09	589	Y	33
*Dc*TPS10	588	Y	34
*Dc*TPS11	551	-	-
*Dc*TPS12	551	-	-
*Dc*TPS13	566	-	-
*Dc*TPS14	647	-	-
*Dc*TPS15	577	-	-
*Dc*TPS16	632	-	-
*Dc*TPS17	551	-	-
*Dc*TPS19	562	Y	37
*Dc*TPS21	551	-	-
*Dc*TPS23	553	-	-
*Dc*TPS25	826	Y	59
*Dc*TPS26	570	-	-
*Dc*TPS27	593	Y	44
*Dc*TPS28	769	-	-
*Dc*TPS29	836	-	-
*Dc*TPS30	609	Y	43
*Dc*TPS32	554	-	-

*Dc*TPS33	593	Y	17
*Dc*TPS38	567	-	-
*Dc*TPS42	541	-	-
*Dc*TPS43	562	-	-
*Dc*TPS44	582	-	-
*Dc*TPS45	517	-	-
*Dc*TPS46	534	-	-
*Dc*TPS47	512	-	-
*Dc*TPS48	587	Y	39
*Dc*TPS52	587	Y	26
*Dc*TPS53	560	-	-
*Dc*TPS54	588	Y	43
*Dc*TPS55	598	Y	34
*Dc*TPS56	804	-	-
*Dc*TPS57	763	Y	78
*Dc*TPS59	772	-	-
*Dc*TPS60	554	-	-
*Dc*TPS62	506	-	-

Supplementary Table S3. qRT-PCR primers used in this study.

**Primer_Name**	**Primer_Seq**	**Tm**	**Amplicon (bp)**
*Dc*TPS01F_int	CAACATCTTCAGCTTCTACC	53.7	281
*Dc*TPS01R_int	CATACGCTGCAACACAGC	57.2	281
*Dc*TPS02F_int	CTAACACTTGTGCTCTAAAACC	55.3	248
*Dc*TPS02R_int	CAGCAGCTCCAGCTTGTC	58.4	248
*Dc*TPS32F_int	GTTTGGGAGGTTCAAAGATG	54.5	391
*Dc*TPS32R_int	CCATGCTCCCTGAGC	52.9	391
*Dc*TPS45F_int	CTTTGAATTTGCGGAAAACTC	54.6	276
*Dc*TPS45R_int	CTTGATCCATTGCCTTATACTGGC	59.8	276
*Dc*TPS46F_int	GGTGAAGAACGTGGTACG	55.5	218
*Dc*TPS46R_int	CGAAGTTGTCAAAAACATCTGC	57.1	218
*Dc*TPS19F_int	GATATGGCAAATATTAAGCGTG	53.8	384
*Dc*TPS19R_int	GCGGCTCAAGTATTCAG	52.5	384
*Dc*TPS25F_int	CCATCCCTCCTCCTCC	53.9	301
*Dc*TPS25R_int	CGACGAATGAAGCTTAGTGTTTTC	58.9	301
*Dc*TPS30F_int	GCATGAGGTTCTCTTCATTG	54.4	292
*Dc*TPS30R_int	CTCACAATTTCCTTCAATCC	52.1	292
*Dc*TPS09F_int	GCTGCACCTCCCCG	55.8	251
*Dc*TPS09R_int	GCATGGATCAGAAGCTTCG	56.5	251
*Dc*TPS04F_int	GTGAGTGTTGGTATGAGTTTTTCTG	58.6	286
*Dc*TPS04R_int	CCTTCAAATCCCCTGC	51.2	286
*Dc*TPS26F_int	CTGCACTAGGAATGATACTGC	56.2	325
*Dc*TPS26R_int	CCACTTGTCATTCTTGTTCAC	55.4	325
*Dc*TPS56F_int	CAGGGGAGAAAAGAAAGAATC	54.2	297
*Dc*TPS56R_int	CTCAAGAAAATGTGTACCCC	53.9	297
*Dc*TPS28F_int	CTTTCACCCAAATTCTTACATAC	53.7	281
*Dc*TPS28R_int	CATCCTTAAGTTGATTTTCCAATATC	55	281
*Dc*TPS14F_int	CACGGTCTTTTTTCCCC	52.8	334
*Dc*TPS14R_int	GCCTATGAACAGAGTCGAG	54.8	334
*Dc*TPS17F_int	CACACACTGCCAGTTC	52.1	291
*Dc*TPS17R_int	GATTTCCCAGTGATTATTATTTTG	52.5	291
*Dc*TPS57F_int	CATTCCACGTCTTCTTTACTTGC	58.3	345
*Dc*TPS57R_int	CAGAAACATTTATATCTCCATCGTC	55.9	345
*Dc*TPS59F_int	GACAATAATTACACAGGATTCG	53.1	326
*Dc*TPS59R_int	CTTGAAGGGAACATAGGTAGTCC	58	326
*Dc*TPS23F_int	GTTAGGCAAGAACTCAAGG	53.3	355
*Dc*TPS23R_int	CTTCATCTTCTATCCTCAGC	52.6	355

*Dc*TPS60F_int	GCTAATAACAAGGTAGGAATCG	54.5	331
*Dc*TPS60R_int	GTTTTCTCACTAGCTTCTTCTCCTTG	59.9	331
*Dc*TPS44F_int	GCCTTGGATCATTTCAGTTTG	55.7	303
*Dc*TPS44R_int	CAATAAGCTCCAACTTTTCCC	55.1	303
*Dc*TPS29F_int	CTCTCCACAGCTACAAAATTC	54.6	335
*Dc*TPS29R_int	CTGGAAAAACGATGGTAAAC	52.8	335
*Dc*TPS16F_int	GCACTTTCCTCAGTTGGG	55.6	289
*Dc*TPS16R_int	CTAAATCATTGATACTCCACGC	55.1	289
*Dc*TPS15F_int	GTTCTCCACCACATAAGGCTG	58.6	307
*Dc*TPS15R_int	CAAAGCTCATGATACGAATC	52.2	307
*Dc*TPS38F_int	GGAACAAAATGCTGGGG	53.2	329
*Dc*TPS38R_int	CGACCATGAAAAGGCC	52.2	329
*Dc*TPS42F_int	CAGTCTCGGCCATTCGC	58.2	319
*Dc*TPS42R_int	CTTAAGATTCGGAAGTAAAGTGC	55.7	319
*Dc*TPS10F_int	CATCTATAATTTTCCCAGTTTCAAC	54.7	330
*Dc*TPS10R_int	CAACTCTCTGCAACTGATC	53.4	330
*Dc*TPS11F_int	GCTAAAACAGGAAGTGAAG	51.4	395
*Dc*TPS11R_int	GAGAGGTGGTAAATTCAAGAG	53.7	395
*Dc*TPS03F_int	CTATAGTCCACATTTCTCCCC	54.9	377
*Dc*TPS03R_int	CTTCAAGGCACGTTCTATTTC	55.3	377
*Dc*TPS07F_int	CATACTGTAACTCGTCGC	52.8	338
*Dc*TPS07R_int	GTTGTTGTCCTTGAACTTGTAG	55.5	338
*Dc*TPS53F_int	GTTAACGCTGCAACTGG	53.8	353
*Dc*TPS53R_int	CTTAGTGCAACATCATGTAAATTATC	55.3	353
*Dc*TPS12F_int	GCTGCCTGTTATTAGACG	52.9	339
*Dc*TPS12R_int	CCATGTTGTCTGAGAAGTC	53	339
*Dc*TPS48F_int	CTCCATTAGTCACCTGCTGC	58.3	282
*Dc*TPS48R_int	GATCTAGCGGCTCAACAG	54.9	282
*Dc*TPS13F_int	GTGTGCCTGAGATTGTTCG	56.7	339
*Dc*TPS13R_int	GTAACCGGAAGCAAAGAG	53.1	339
*Dc*TPS21F_int	CAGGCGTTCGGGGAATTAC	58.6	361
*Dc*TPS21R_int	CATAAAACTCTCGAATATATCTTC	51.6	361

Supplementary Table S4. Primers for gene cloning.

Primer_Name	Primer_Seq	Purpose
*Dc*Actin_F	CGGTATTGTGTTGGACTCTGGTGAT	Housekeeping gene
*Dc*Actin_R	CAGCAAGGTCAAGACGGAGTATGG	Housekeeping gene
*Dc*PP2A_F	GTGTATCAATGTACCACCAGCAACT	Housekeeping gene
*Dc*PP2A_R	GCTCACCAAGGAACATGACTTCTT	Housekeeping gene
Tubulin_F	TCTTGGAGGTGGCACAGGAT	Housekeeping gene
Tubulin_R	ACCTTAGGAGACGGGAACACAGA	Housekeeping gene
*Dc*TPS1_F	ATGTCTCTCAATGTTCTGGC	Control TPS
*Dc*TPS1_R	TGATGGAACCCGATCAATGA	Control TPS
*Dc*TPS19_F	GGATCCATGTTTAAACAGGCACAAG	Cloning
*Dc*TPS19_R	CTCGAGTCAACAATTTAAAGGTAAAAC	Cloning
*Dc*TPS25_F	GGATCCATGACTGCAGTTTCAAG	Cloning
*Dc*TPS25_R	CTCGAGTTACATCCTACGGCC	Cloning
*Dc*TPS23_F	GGATCCATGGCGATTGTTAGG	Cloning
*Dc*TPS23_R	CTCGAGCTAGAACAATTGTTCCC	Cloning
*Dc*TPS16_F	GGATCCATGGCTGCAGTACTG	Cloning
*Dc*TPS16_R	CTCGAGTTAACCAAGAGTTAC	Cloning
*Dc*TPS04_TCF	GGATCCATGATGGAAAGAGTTTCTTG	Cloning
*Dc*TPS04_TCR	CTCGAGTCAGGTGATCGAAAGAGGTTC	Cloning
*Dc*TPS05F_TC	GAATTCATGAGTACTCTTATTGTGAATC	Cloning
*Dc*TPS05R_TC	GTCGACTTACAGAAGAACAGG	Cloning
*Dc*TPS03F_TC	GGATCCATGGGTTCTCAATCC	Cloning

*Dc*TPS03R_TC	GTCGACTCATATATCGATGGGGTC	Cloning
*Dc*TPS07R_TC	GGATCCATGAATTCTACTTCTGG	Cloning
*Dc*TPS07F_TC	CTCGAGTTATATAGGAAAAGGGTC	Cloning
*Dc*TPS53R_TC	GGATCCATGGCTATGTATGTTAACG	Cloning
*Dc*TPS53F_TC	CTCGAGTTATGCTGGAATCGGATC	Cloning
*Dc*TPS03B_TCF	GGATCCATGTCCATGGGAATTTCTG	Cloning
*Dc*TPS03B_TCR	GTCGACTCAGTCAATATCGATGGGG	Cloning
*Dc*TPS53B_TCR	CTCGAGTTACCCTGAAATCGGATC	Cloning
*Dc*TPS48_F	GGATCCATGGAGGGATCTGTGTCACCAG	Cloning
*Dc*TPS48_R	GTCGACTCATATAGGCTCAACAAGAAGGG	Cloning
*Dc*TPS14_R	CTCGAGTCACTCATTAAGAGTGAAGG	Cloning
*Dc*TPS42_F	GAGCTCATGAGCAGCCAGTCTCG	Cloning
*Dc*TPS42_R	GTCGACTCATATCGGTATCGGATCCACC	Cloning
*Dc*TPS11_F	GGATCCATGGCTTCAAGTGTGGG	Cloning
*Dc*TPS42_R	CTCGAGTTATGCTGGAATGGGATTTATG	Cloning
*Dc*TPS10_F	CTCGAGATGGCCGCTGAAACAAC	Cloning
*Dc*TPS10_R	GTCGACTCAGAGAGGGATGGGCTC	Cloning
*Dc*TPS30_F	GGATCCATGTGCAGTAGCAGTAGTCATG	Cloning
*Dc*TPS30_R	CTCGAGCTATAGAGGTATGGGGTCAAC	Cloning
*Dc*TPS15_F	GAGCTCATGTCTGTGTTTCTTCAGGC	Cloning
*Dc*TPS15_R	GTCGACTCATATTGAGATGGGATCCACTAG	Cloning
*Dc*TPS38_F	CTCGAGATGTCGGCTTCAGCAGG	Cloning
*Dc*TPS38_R	GTCGACTTAGACAAGTTGTGGCATTG	Cloning
*Dc*TPS28_F	GGATCCATGGCCTCACTGGAGTC	Cloning
*Dc*TPS28_R	CTCGAGCTATACTCGGGTAATAGGTTTG	Cloning

Supplementary Table S5. Terpene compounds identified from random forest analysis and Boruta variable selection of colored carrot root tissue. ANOVAs comparing the concentrations of the selected compounds among groups are reported (F-statistic and p value). All compounds were identified by comparison to authentic standards or compounds in an Opopanax essential oil except bornyl acetate, which was identified by comparison to mass spectral libraries.

Compound	Boruta IMP Factor	F Value	p Value
α-pinene	4.7437306	3.6368	6.40E-02
β-pinene	4.9868365	4.7613	3.448e-02*
α-terpinolene	7.2058394	4.1936	4.659e-02*
bornyl acetate	8.1511563	20.344	4.228e-04***
(*E*)-β-farnesene	8.003681	21.099	3.721e-04***
(*E*)-β-caryophyllene	7.2949134	42.36	2.955e-05***
α-humulene	5.5921902	30.176	1.034e-04***
β-bisabolene	4.4540192	12.795	2.022e-03**
(*E*)-γ-bisabolene	6.7570118	8.3796	7.506e-03**

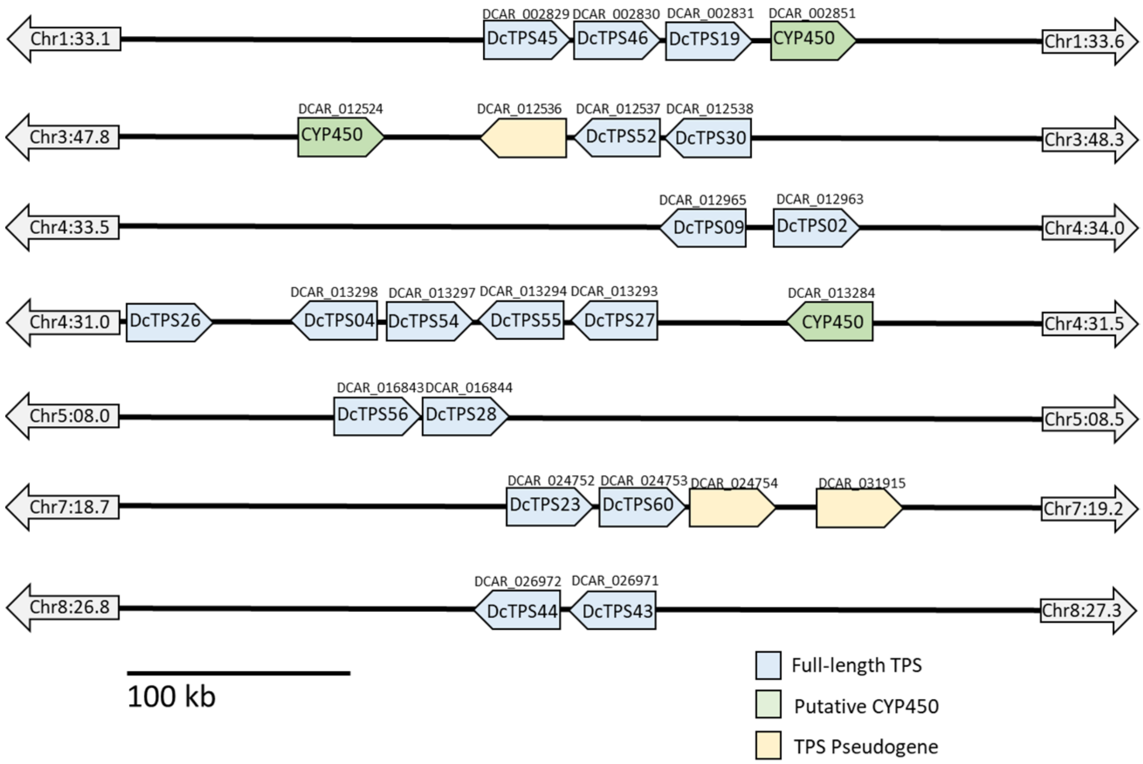

Supplementary Figure S1. Diagram of 7 gene clusters identified in the DH1 carrot genome. Full-length TPSs are highlighted in blue and cluster by TPS sub-family. TPS pseudogenes are highlighted in yellow and putative cytochromes P450 clustered in the similar chromosomal region are listed. Chromosomal positions are indicated by gray arrows in Mbp.

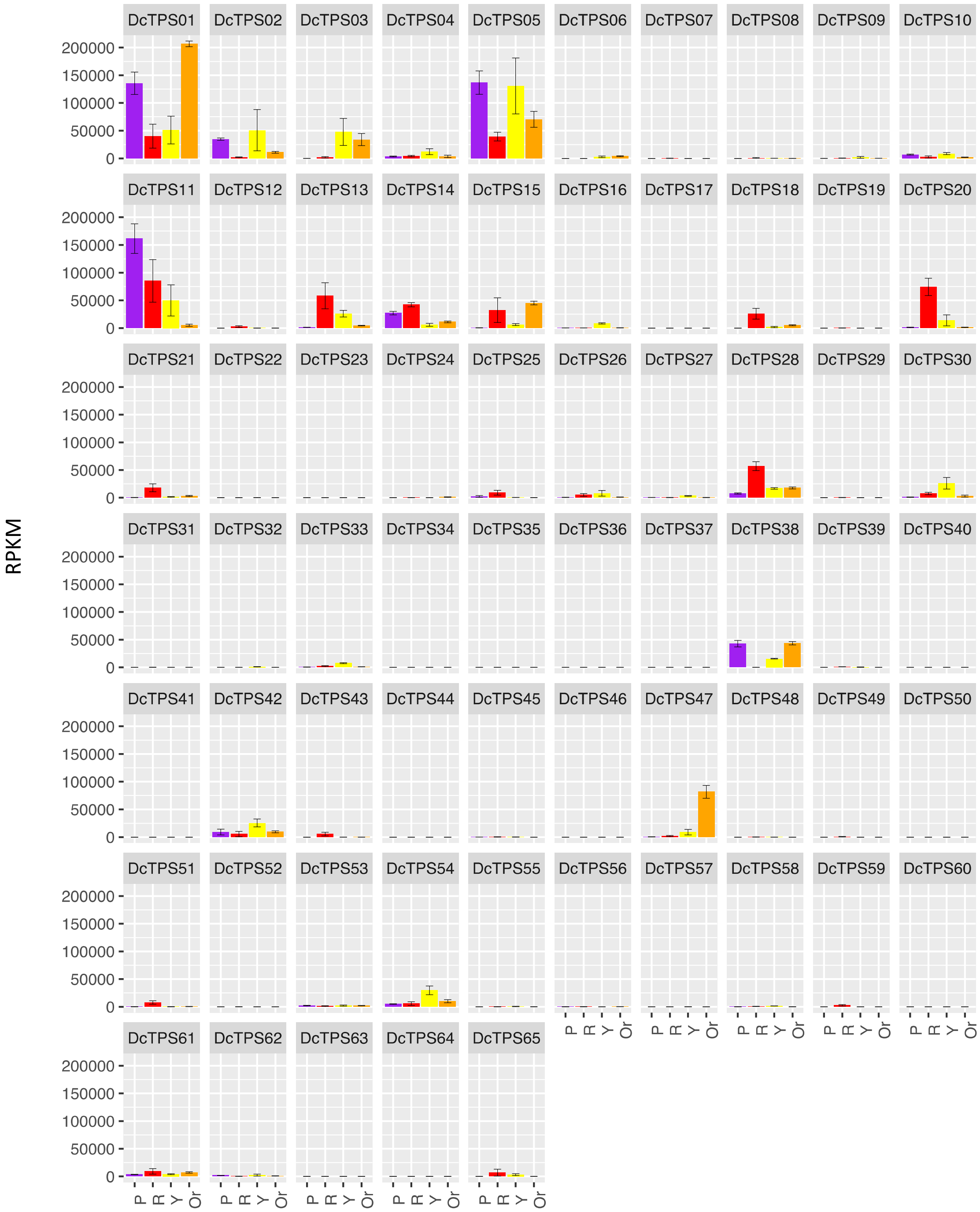

Supplementary Figure S2. RNA-seq analysis of the 65 TPS gene models predicted by Keilwagen et al.[19] across four colored carrot varieties. Gene expression is presented as reads per kilobase of transcript per million mapped reads (RPKM). P: purple (cv. P7262), R: red (cv. R6637), Y: yellow (cv. Y9244A) and O: orange (cv. B493B).

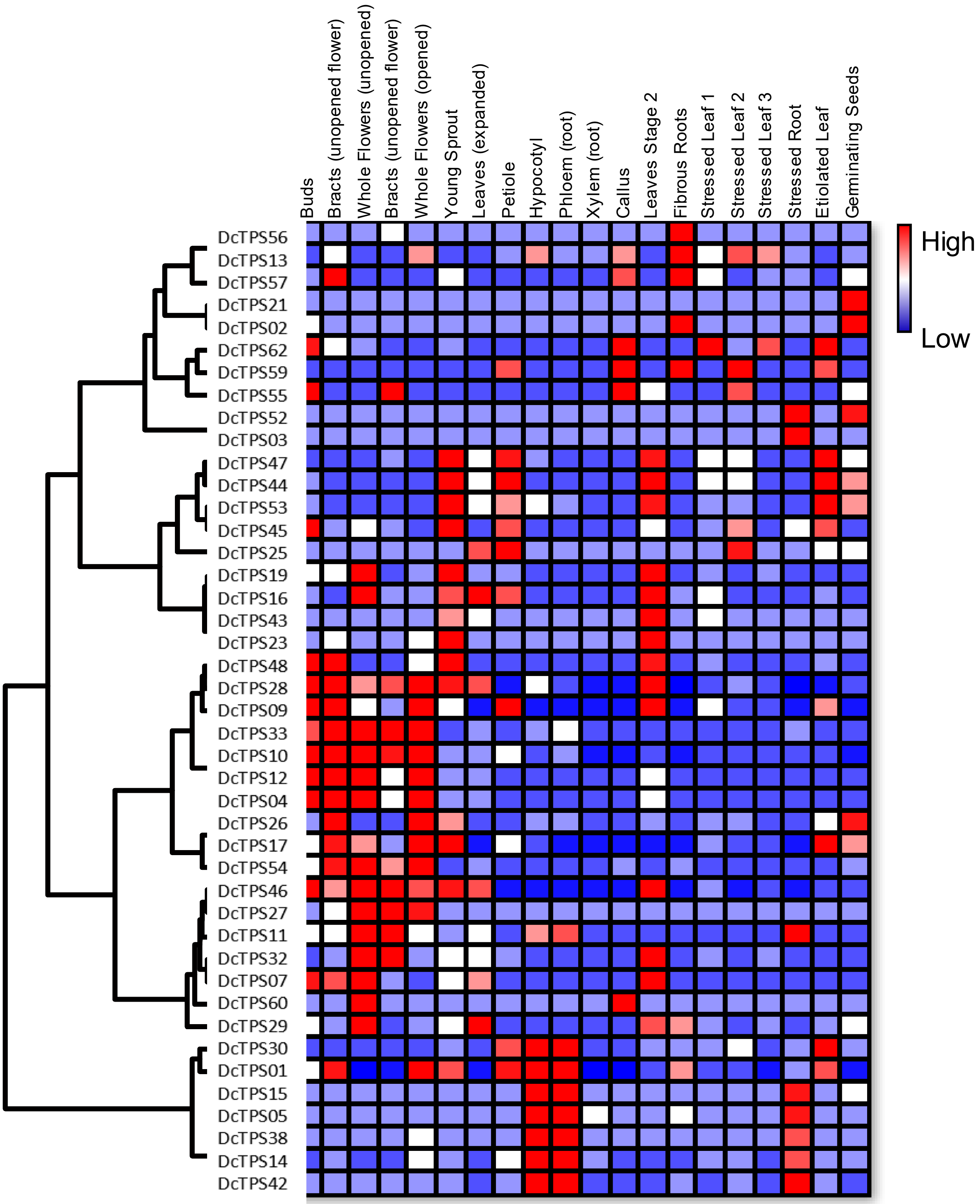

Supplementary Figure S3. Hierarchical cluster analysis of TPS gene expression profiles across DH1 carrot tissues. Heat map comparing relative transcript abundance for all 43 TPS genes candidate in FPKM (Fragments Per Kilobase of transcript per Million mapped reads) in 20 tissues. Specific gene expression data sets are from Iorizzo et al.[18].

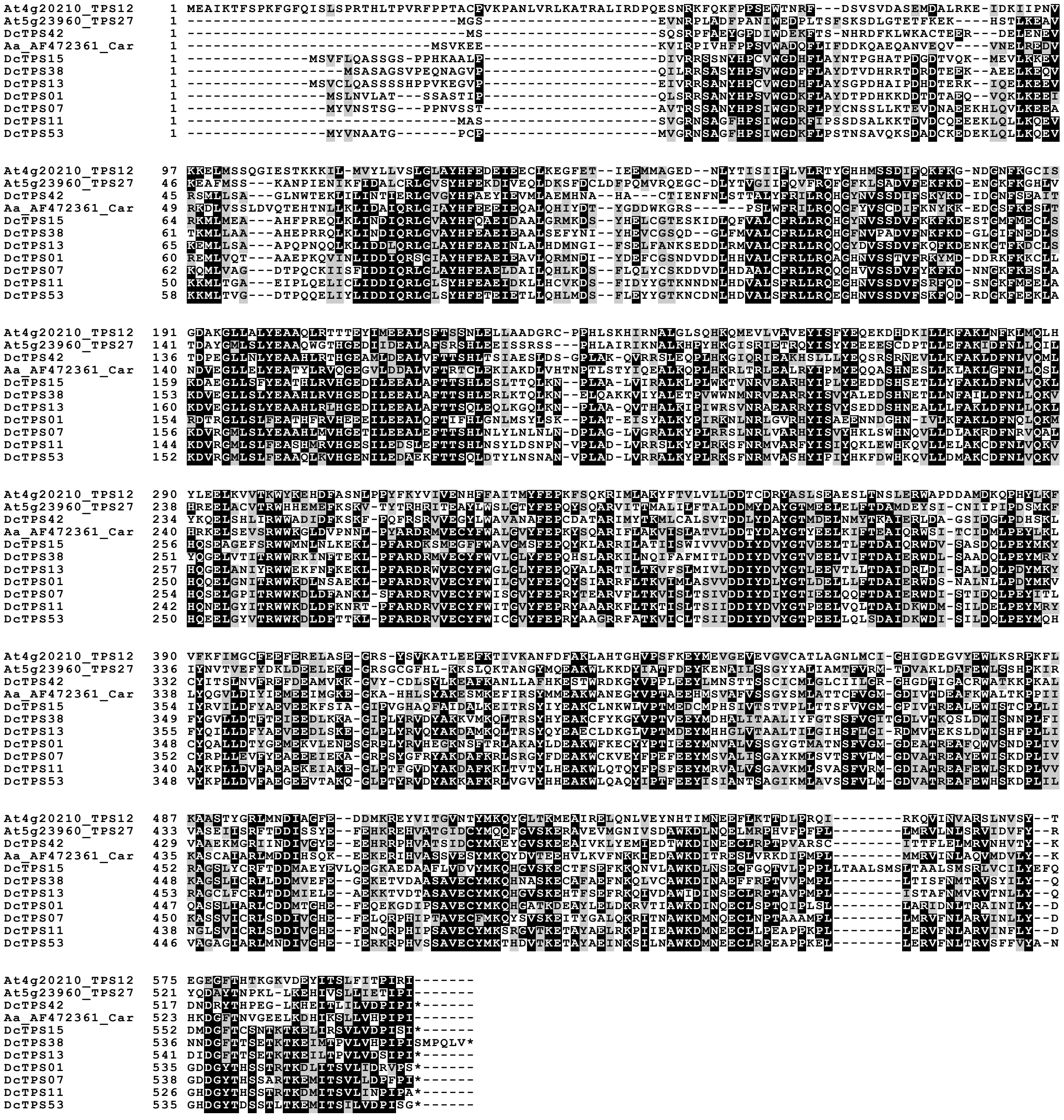

Supplementary Figure S4. Amino acid sequence alignment of proteins in the carrot TPS-a sub-family and other select TPSs of this subfamily. Alignments were performed using Clustal W[43] and visualized with BoxShade (v3.21, https://embnet.vital-it.ch/software/BOX_form.html); At, *Arabidopsis thaliana*; Aa, *Artemisia annua*; Dc, *Daucus carota*.

```
DcTPS16           1 ----------------------------MAAVLHALSSVGRQCSLFETDILDAPRFSNYLHKPGRVQMAGLQFIATRRHEKCQSCYTSNR
At_myrcene_s      1 ---------------------------------------------------------MATLCIGSAPIYQNACIHNFRLQRPRRFISKSM
DcTPS14           1 MPIFKLRRSQLIFNYFFHLLERCITEYIWCNLCENKIYNKLLTYRIQKRSFSCQTIPKQMALHGLFSPFLVTAPSRMAIPSSNAPKLCAS
DcTPS09           1 ---------------------------------------------------------MVMAVQGLFSSFLLAAPPRI-PPLPFARTGSKI
DcTPS32           1 -----------------------------------------------------------------------MCLGGSKMTR---------
DcTPS33           1 -----------------------------------------------------------MALKGLSSTLLVTALPRSSVPSGRNH-NKSF
DcTPS02           1 -----------------------------------------------------------MALPALFSTFIVTAPPRTSLSLARNP--SNT
DcTPS62           1 -------------------------------------------------------------------------MPATSVVIVH-------
Sf_cineole_s      1 ----------------------------------------------------MSSLIMQVVIPKPAKIFHNNLFSVISKRHRFSTTITTR
Mc_linalool_s     1 ----------------------------------------------MCTIISVNHHHVAILSKPKVKLFHTKNKRSASINLPWSLSPSSS
So_bornylPP_s     1 ------------------------------------------------------MSIISMNVSILSKPLNCLHNLERRPSKALLVPCTAP
DcTPS26           1 -------------------------------------------------------------------------------MHTTSSHCTRN
DcTPS55           1 -----------------------------------------------------MSSSATRFMVKPAASLTLLQPPSTKAIHTTSTCCTSN
DcTPS54           1 ------------------------------------------MALISTVSLGMSFSATRF-MVKPAASLTLVKPPSTKSIHTTSIRCTSN
DcTPS04           1 -------------------------------------------MVLVSTVSVGMSFSATRFMVKPAASFTLLKPPSTKAIHNTSIRSTSN
DcTPS27           1 ------------------------------------------MAVISTVSLGVSISATRLILLKPASSLTHLKPPSTKAIHTTSIRCTGN
DcTPS30           1 ---------------------------------------------MALSMVNLGMRFSSLHSMLKPTSFTCVNPSSNAKKVFEVPGIRCS
DcTPS52           1 -----------------------------------------------------------------------MLKPTSFASVNRSFTAGKV
DcTPS17           1 ---------------------------------------------------------------------------MNRLLADHTL-----
DcTPS21           1 ---------------------------------------------------------------------------MSTLIADQIF-----
DcTPS05           1 ---------------------------------------------------------------------------MSTLIVNHSL-----
DcTPS12           1 ---------------------------------------------------------------------------MSTLIAKHQL-----
DcTPS03           1 ------------------------------------------------------MAASTIVHISPLTIGSQSTLPRLTPGITKSVTACKS
DcTPS48           1 ---------------------------------------------------------MASAFILNSPLVTCCNFRRSLPCKPKFLTLSKS
DcTPS10           1 ------------------------------------------------------------MASIIFPVSTLINFQRFTTCKPKAATACKS
DcTPS47           1 ---------------------------------------------------------MWKLEKFRFSDFVLMDNHIHTLIVQLASC----

DcTPS16          63 ESIQPVSAASESQATVIPAASAEVASAQSVSAPAWSINDLAAQLKFKNYKQDEVYEQMATKLEIEIRMTIEDEMASNPLNFFELIDNIER
At_myrcene_s     34 TKTMPDA-----------NPLDLRRRSGNYQPSSW-DHSYLLSIENK-YV-NEKEVITRHVLKKKVKKMLEEVETKSRLEKLELIDDLQK
DcTPS14          91 K---PVQCITTPVTTTDHDQGSASRRNANYVPSFW-DYNVVKSLSSN-YD-EKKYEMQVEELKEDVKRLIHA-ETDVPLAKLELLDSVHR
DcTPS09          33 CATKPVQCIKTTDVP---HQGSGSRRNANYPPSFW-DYNLIKSLSSD-YN-EEKYVKQVDELKDDAKLLIHA-DTETPLAKLELLDSVQR
DcTPS32          11 ------------------DSGAALRRNANYPPSSW-DYNFVKSLNSD-FT-EEKYARQLDELKDDVKRLIYA-ETDVPLAKLELLDSVQR
DcTPS33          31 ATERSVQCIKTTATIIDQDSGAALRRNANYPPSSW-DYNFVKSLTSD-YT-EEKYARQLDELKDNVKRLIHAETDDP-LAKLELLDTVQR
DcTPS02          30 CALKPVQCIMTNPKTNDPDQGTPSRRNANYAPSFW-DYSFVKSLSSD-YA-EEKYVKQVDELKDDVKSLIHA-ETEV-LDKLELLDSVQR
DcTPS62          11 -----------------------------YQLQLF---ILIYQLNVIYFS-GSKICKAG-------------------------------
Sf_cineole_s     39 GGRWAHCSLQMG------NEIQTGRRTGGYQPTLW-DFSTIQLFDSE-YK-EEKHLMRAAGMIAQVNMLLQE-EVDS-IQRLELIDDLRR
Mc_linalool_s    45 AASRPISCSISSKLYTISSAQEETRRSGNYHPSVW-DFDFIQSLDTDHYK-EEKQLEREEELIMEVKKLLGA-KMEA-TKQLELIDDLQN
So_bornylPP_s    37 TARLRASCSSKL------QEAHQIRRSGNYQPALW-DSNYIQSLNTP-YT-EERHLDRKAELIVQVRILLKE-KMEP-VQQLELIHDLKY
DcTPS26          12 DTALVNNDTACV------DDKSIVRRSGNFPPPIW-DDDFVQSLTSD-FK-GEICNKYAGDLKERVRMLLNKEDTDN-LKKLELVDSVQR
DcTPS55          38 DAALVSRGDACV------DDKSIVRRSGNYPPPIW-DDDFVQSLASD-FK-GEICNKYAGDLKERVRMLLNKEDTDN-LRKLELVDSVQR
DcTPS54          48 DTALVSRGDACV------DDKSIVRRSGNYPPPIW-DDDFVQSLDSD-F-------------KERVRMLLNKEDMDN-LKKLELVDSVQR
DcTPS04          48 DAALVSRGDACV------DGKSIVRRSGNYPPPIW-DDDFVQSLASD-FK-GEIFNKYAGDLKERVRMLLNKEDTDS-LKKLELVDSVQR
DcTPS27          49 DTALLSRGDACV------DDKSIVRRSGNYPPPIW-DDDFVQSLASD-FK-GEICNKYAGDLKEHVRMLLNKEDMDN-LKKLELVDSVQR
DcTPS30          46 SSSHDSAVVSTGNVS---DEVSDGRRSGNYPPSMW-DYDFFQSLSSD-FK-GEICNKKANGLKEIVRMLLDKEDLDS-LHKLELIDTIQR
DcTPS52          20 VKISSIRCTSTR------DKISIGRRSGSYSPPTW-DYDFFQSLASS-FK-GEICSRQACELKENVRFLLNKEDLDS-LHKLELVDAIQR
DcTPS17          11 ---------------------PVLRRSGNYKPCIHRHNNLVQSLTTE-YK-IERFKGRVDKLREDVVEMFND-VSEP-LDQLELIDDLQR
DcTPS21          11 ---------------------PVTRRSGNYKPCVHRDNNLVQSLNTD-FK-VERFKERVDELKEEVVGMFAD-IIKS-LDQLELIADLQR
DcTPS05          11 ---------------------PVVRRSANYEPCIW-DADFVQTLATD-YT-GERFNGRVNELKGNVIGMLND-VAKPNLDRLELIDYLQR
DcTPS12          11 ---------------------PVIRRSANYQPCIW-TNDFVQSLTTD-YT-GERFNGRVNELKENVIEMLND-VAKPNLDQLELIDNLQR
DcTPS03          37 VLIRSSMGISV-------TPEPIIRRSGNYQPCMW-DNKFLQSLKSD-YT-GEGSKEPASELKEVVRFMFKN-VVEP-LDQLEFIDHLQR
DcTPS48          34 VVEFVTGNEGSV------SPEPIRRRSGNYKPCMW-DNNFLQSLKTN-YT-GEPINARASMLKEEVRLMFDD-AVEP-LDQLELIDLLQR
DcTPS10          31 VITSAAETTKLPV-----PAEPIVRRSANYKPCLY-DNNFLQSMKTE-FK-GEAVDARASELKEEVRMIFNN-VAGP-LEQLELIDQLQR
DcTPS47          30 --------------------------------HMW---KLKKKNRFSDF--GEAIDARASELKEEVRMIFKN-VAEP-LDQLELIDQLQR

DcTPS16         153 LGLGYRFEKDISTALTK--YVSLEGAPEYHN----SLHSTALRFRLLREHGYKVSQ--------DVFQSFKDE-NG-AFMPSL-LADVKG
At_myrcene_s    110 LGVSYHFEQEINNILTN--FHLENGKNIWKCDKEEDLHATALEFRLLRQHGFGVSE--------DIFDVIIDKIESNTFK----SDNITS
DcTPS14         174 LGLNYQFQKDIKQAIDRI-----CEADSQLGN---DLHSTALLFRILREHGYTVSQ--------DVFQKFTDE-TG-KFKANL-GEDVKG
DcTPS09         116 LGLKHLLKKDIKQAVDAI-Y--NNSVDAWLSD---DLHSTALRFRILREHGYAVSP--------DVFLRFTNE-EG-NFKENL-CGDVKG
DcTPS32          79 LGLNYQFQNDVKQAVDVI-Y--NNISDASLSD---DLYTTALQFRMLREHGYTVSQ--------DVFGRFKDD-TG-NFKANL-CEDVKG
DcTPS33         117 LGLNHRFQNDVKQAVDVI-Y--NNINDAWLSD---DLYTTALQFRMLREHGYTVSQ--------DVFGRFKDD-TG-NFKANL-CEDVKG
DcTPS02         115 LGLKYQFQKDIMQAVDVIHNNNNNSADAWLSD---DLYLTALKFRILREHGHTVSQ--------DVFQRFIDDTAG-KFKANL-CGDVKG
DcTPS62          37 -GLKYQFQEDLKQAVDVI-Y--NDSADAWLSD---DLYSTALKFRIFRDYGYTVEQ--------DVFQKFRDS-TARNFKANL-CEDVKG
Sf_cineole_s    118 LGISCHFDREIVEILNSKYYTNNEIDES-------DLYSTALRFKLLRQYDFSVSQ--------EVFDCFKND-KGTDFKPSL-VDDTRG
Mc_linalool_s   131 LGLSYFFRDEIKNILNSI-YKIFQNNNSTKVG---DLHFTSLGFRLLRQHGFNVSQ--------GVFDCFKNE-HGSDFEKTLIGEDTKG
So_bornylPP_s   116 LGLSDFFQDEIKEILGVI-YNEHKCFHNNEVEKM-DLYFTALGFRLLRQHGFNISQ--------DVFNCFKNE-KGIDFKASL-AQDTKG
DcTPS26          92 LGVGHHFKNEIIRILEAV-----NKNDKWDGKD--NLQATSLKFRLLRQHYFKVPQ--------EVFNGYLNK-SG-KFKISL-AGDMKG
DcTPS55         118 LGVGYHFKNEINRILEAV-Y----NNEKWYGKD--NLHATSLKFRLLRQHYFKVPQ--------EAFSGFLNE-SG-KFKISL-TSDMKG
DcTPS54         116 LGVGYHFKNEITRILEAV-Y----STEKWDGQD--NLHATSLKFRLLRQ---------------QAFSGFLNE-LG-KFKISP-TSDMKG
DcTPS04         128 LGVGYHFKNEINRILEAV-Y----STEKWDGQD--NLHATSLKFRLLRQHYFKVPQ--------EAFNGFLNE-SG-KFKISL-TSDMKG
DcTPS27         129 LG----------------------NNEKWDGQD--NLHATSLKFRLLRQHYFKVPQ--------EAFNGFLNE-SG-EFKISL-TSDMKG
DcTPS30         129 LGVSYHFEDEIKRILEAI----HIADEKLSSE---ELNATSLKFRLLRQHNFSVPQ--------EIFEEFMDE-SG-KFKASL-SKDMKS
DcTPS52         100 LGVSYHFEDEIKRILEAI-YNNDEKLNSQ------DLHATSLKFRLLRQHNFRPCSRVFLGEKANIFKQFMDE-SG-KFKASL-RKDMKG
DcTPS17          76 LGVAYYFDGEIKSTLEKI-FE-DQNNNHWEIK---DLHATALKFRLLRQHGYAVSE--------DVLESFMEN--G-SIKECL-WDDVKG
DcTPS21          76 LGLANHFDEEIKRTLKKI-YE-NRTSDLWEIK---DLHATALMFRLLRQHRFDISE--------DIFESFMVN--G-SFKEGL-GDDVKG
DcTPS05          76 LGLGYHFEQEIKSALTKM-YE-DPSYETLERN---DLHGAALRFRLLRQHGFNISQ--------NVFEYFMEN--G-SFKGCL-CEDAKG
DcTPS12          76 LGLGYHFEHEIKSILMKI-YEDDQSSETLERN---DLHAAALKFRLLRQHGYDISQ--------DVFKYFMEN--G-SFKGCL-CNCFKG
DcTPS03         115 LGVAYHFFDEIERALKKI-HSDDTNNDKWER----NLHATALRFRLLREHGYDVSQ--------EDFKGFTEN--E-RFKESL-DEDVKG
DcTPS48         113 LGLAYHYEEEIQRTLKKI-QNGEINVDDREK----DLHATALEFRLFRQHGLFISP--------EGFKQFMSN--G-NFMCS--GADIKG
DcTPS10         111 VGVDYHYRDEINRALKSI----HQNAETWEK----DLHATALEFRLLRQHGHYISP--------EGFNKFTEN--G-SFNKSI-HSDVRG
DcTPS47          81 VGLDYHFQDEIKHALKSIQHNGQNSETTSDK----NLHATALEFRLLRQHGHYISS----------------------------------
```

Supplementary Figure S5. Amino acid sequence alignment of proteins in the carrot TPS-b subfamily and other select TPSs of this subfamily. Alignments were performed using Clustal W[43] and visualized with BoxShade (v3.21, https://embnet.vital-it.ch/software/BOX_form.html); At, *Arabidopsis thaliana*; Dc, *Daucus carota*; Mc, *Mentha citrata*; Sf, *Salvia fruticosa;* So, *Salvia officinalis.*

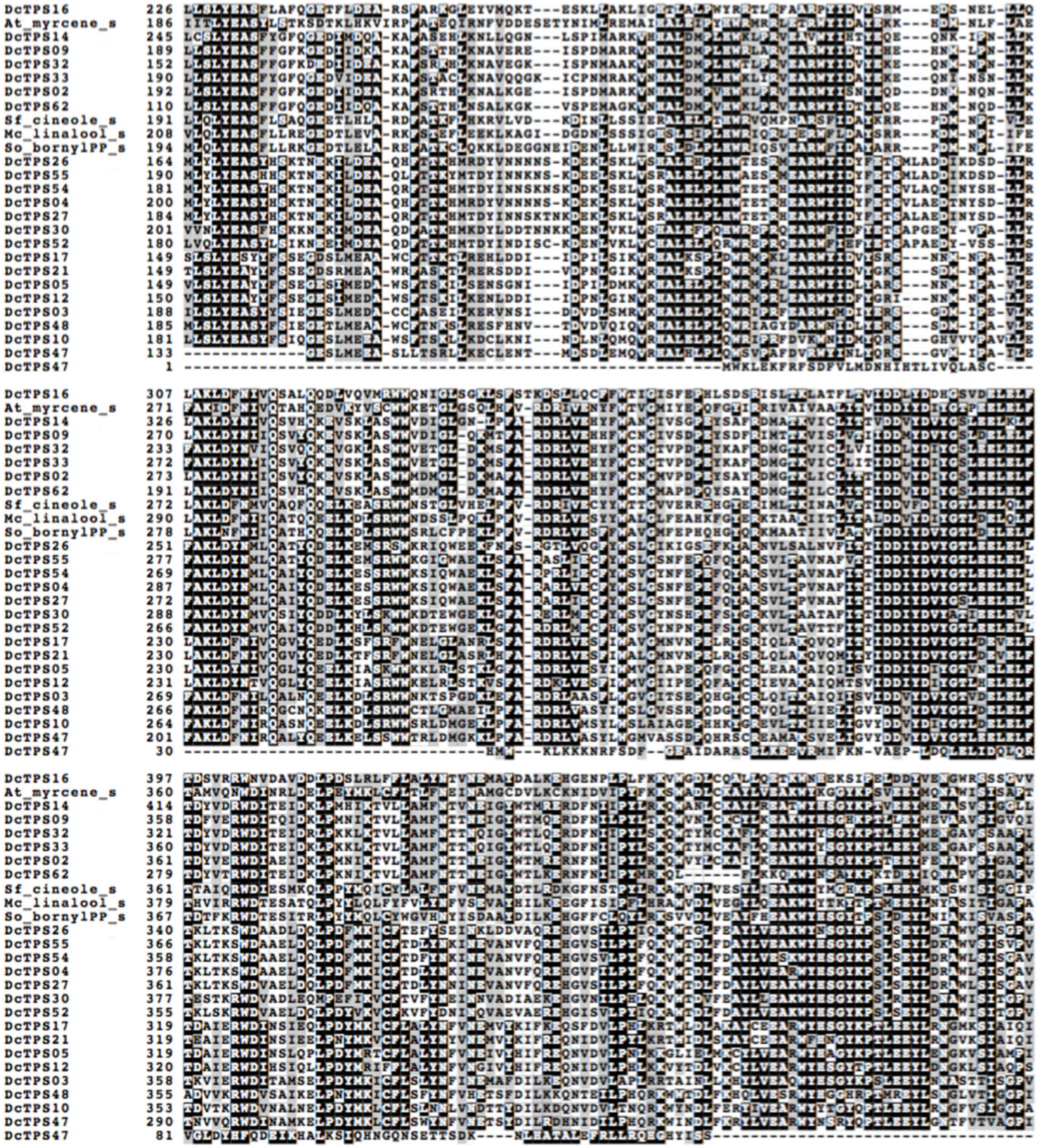

Figure S5. Continued

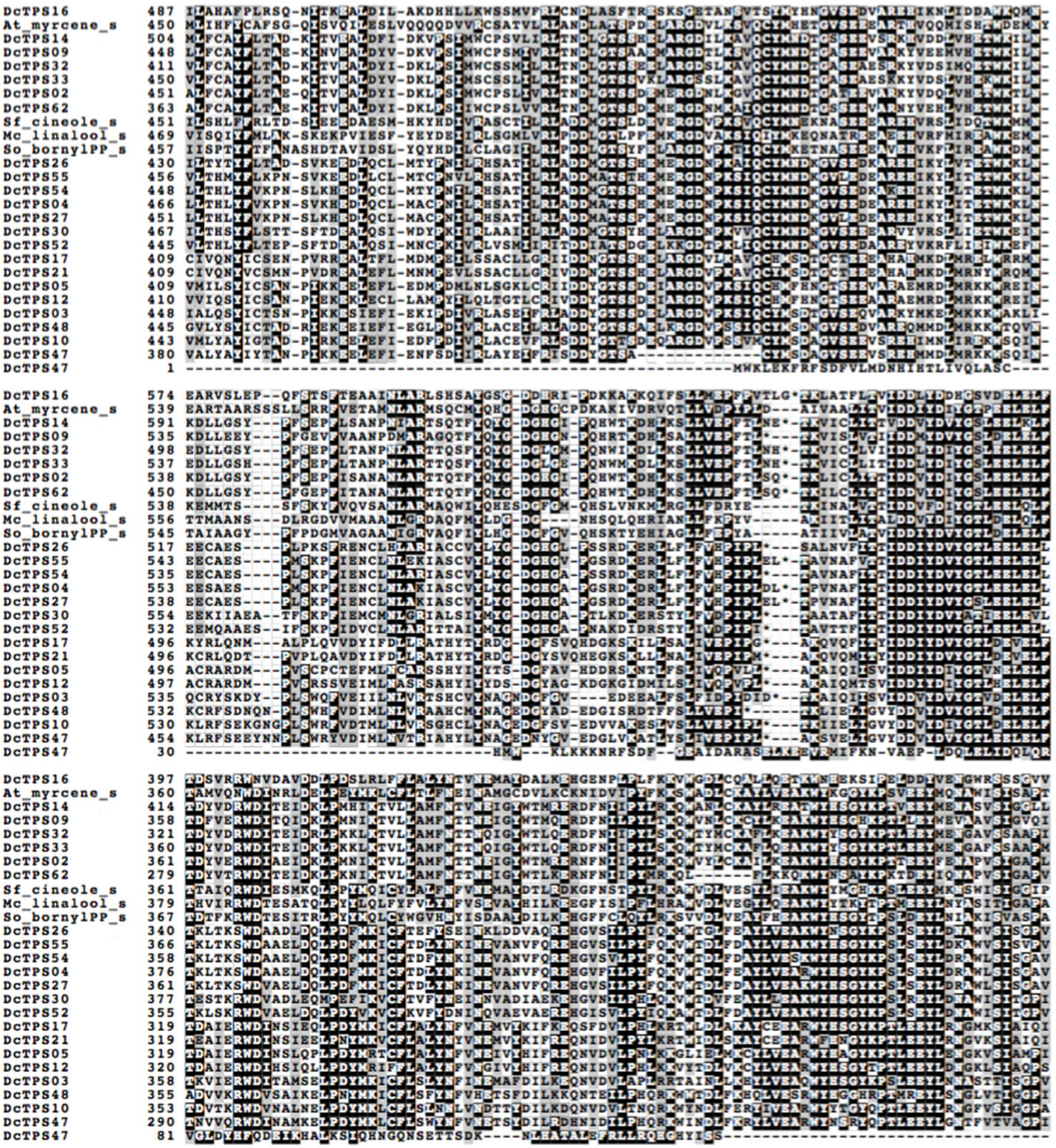

Figure S5. Continued

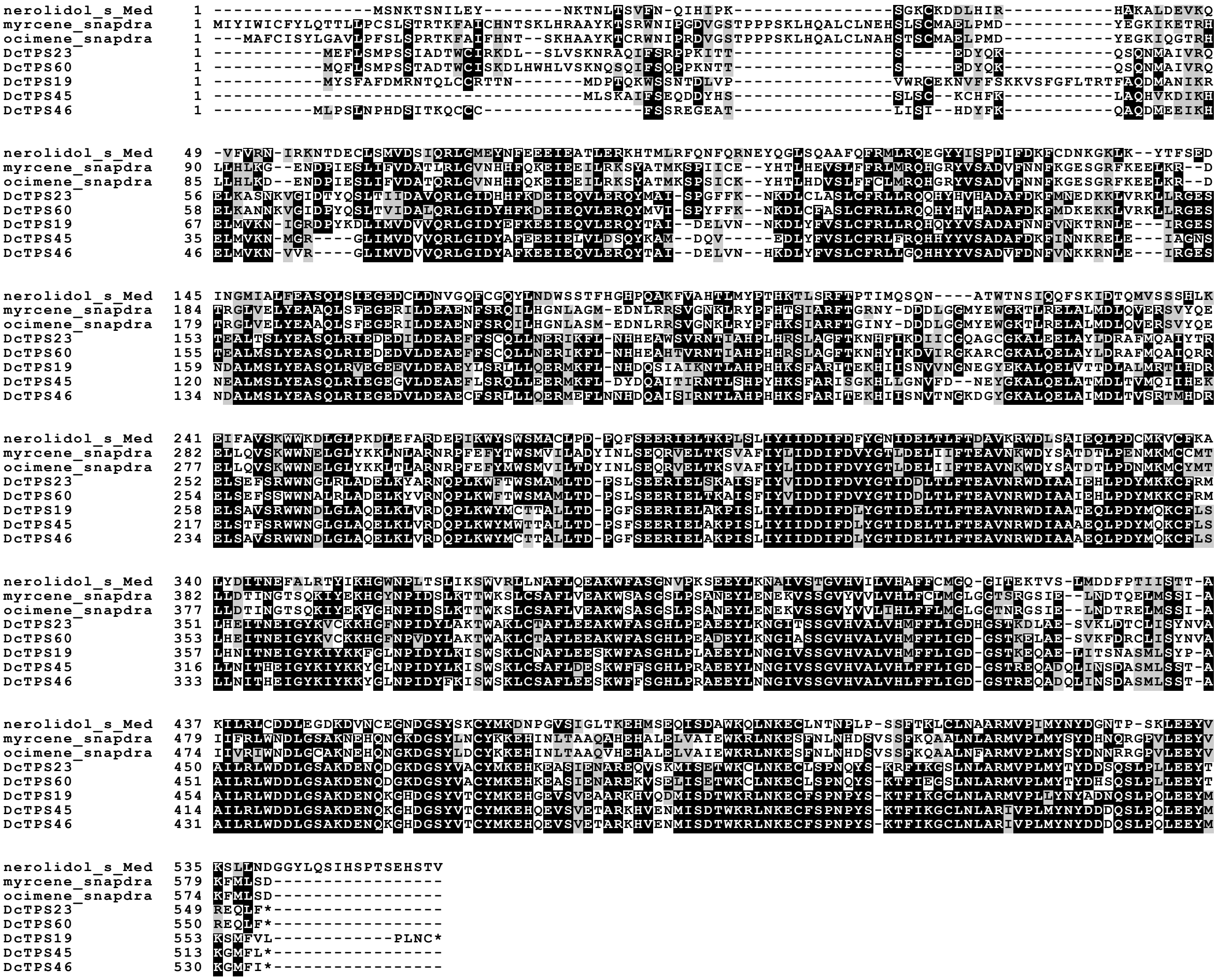

Supplementary Figure S6. Amino acid sequence alignment of proteins in the carrot TPS-g sub-family and other select TPSs of this subfamily. Alignments were performed using Clustal W[43] and visualized with BoxShade (v3.21, https://embnet.vital-it.ch/software/BOX_form.html). Dc, *Daucus carota*; Med, *Medicago truncatula*; snapdra, *Antirrhinum majus*.

```
AT4G02780.1    1 -------------------------------------------------------------------------------------------MSQEVQHDL
DcTPS59        1 --------MRMALCIHINSTNQN-------RSFLSTIIT--------QVLQENLHHRFSTVVFYVARINLVVFVVYFECEFA----------DLAVQNKL
DcTPS25        1 -----------MSSVYHPSSSPS------LQKFPPATFTRPSVSV--SVSVPNSPNHFSGPNAVKGKDIWVKLRFGVQCTAVSRSRTTEYEYAESLKSDL
DcTPS57        1 MLRHLQQTITMYSSTSTKTMSPSHSTSSLLAEFISPTFTSPPLLFHPQKLFCNIRDLAFSVDSVNGQDKRVKLRYSAQCSAVSRSHT-----ATDVEKNL

AT4G02780.1   10 PLIHEWQQLQGEDAPQISVG-SNSNAFKEAVKSVKTILRNLTDGEITISAYDTAWVALIDAGDKT--PAFPSAVKWIAENQLSDGSWGDAYLFSYHDRLI
DcTPS59       68 PALVNWQKILEPHKIRKTDK-RCFSPW-------------TTELVTTISAYDTAWVALVEDSDKTGLPMFPSSLEWIADNQLSDGSWGDDKLFLAHDRIL
DcTPS25       82 PAVINWQEILESDKKGENTKLHSSIKIIKCINSIREMFRSMDDGAISLSAYDTAWVALVEDMNEPGVPQFPASLQWIVSNQLPDGSWGDDKLFLAHDRIL
DcTPS57       96 SSVIYWQEILESHKQEEDTKIREFTKL------IREIFNSMDDGDINVSAYDTAWVALVEDINKTGIPQFPSSLQWIADNQLPDGSWGDKNVFLAYERIL

AT4G02780.1  107 NTLACVVALRSWNLFPHQCNKGITFFRENIGKLEDENDEHMPIGFEVAFPSLLEIARGINID-VPYDSPVLKDIYAKKELKLTRIPKEIMHKIPTTLLHS
DcTPS59      154 NTLACVVALKTWTVHPQKMERGLLFIRENINKLADVKIEHTTMGFEIVFPSLVEVAESLNIE-IPKNLPIMKEIYAQRDLKRSRIPKDKMHEVPTTLLYS
DcTPS25      182 NTLACVIALKSWNVHPEKMERGLLFINENISKLEDEEMEHMPIGFEVAFPSLVEIAETLNIQ-IPKDLPILQEIYAQRDLKLSRIPKDIMHKVPTTLLHS
DcTPS57      190 CTLACVVALRTWNIHPQNMERGLLFIRENINKLEDDEMEHMTIGFEVAFPSLVEIAETLNIKVISKDLPIIQAIYAQRDLKLS-----------------

AT4G02780.1  206 LEGMRDLDWEKLLKLQSQDGSFLFSPSSTAFAFMQTRDSNCLEYLRNAVKRFNGGVPNVFPVDLFEHIWIVDRLQRLGISRYFEEEIKECLDYVHRYWTD
DcTPS59      253 LEGMVDLKWEKLLKLRCEDGSFLSSPSSTAYALMQTKDAKCFDYLSKAIQKFNGGVPNTYPVDMFEHNWVVDRLERLGISRHFKPEIKECMDYVVYRYWTK
DcTPS25      281 LEGMAEMEWEKLLKLQCEDGSFLFSPSSTAFALMQTKDENCLNYLSRIVRKFDGGVPNVYPVDMFEHIWVVDRLERLGISRYFKSEIKECMDYIHRYWTN
DcTPS57      273 ----------------------------------------------------NSVPNAYPVDMFEHCWAVDRLERLGVSRYFKYEIKDCMDYVVYRYWTS

AT4G02780.1  306 NGICWARCSHVQDIDDTAMAFRLLRQHGYQVSADVFKNFEKEGEFFCFVGQSNQAVTGMFNLYRASQLAFPREEILKNAKEFSYNYLLEKREREELIDKW
DcTPS59      353 KGICWGRTLNITDIDDTAMAFRLLRLHGYSVSPDVFRNFESNGKFFALAGESNQAVTGMLNLLRVSQVSFPEESLLSAAKKFSTDFLRSKRADGQLLDKW
DcTPS25      381 QGICWARNTRVNDIDDTAMAFRLLRLHGYTVSPDVFKNFESKGEFVCFAGQSNQAVTGMFNLLRASQVVFPEEALLEEAKKFSIEFLRGKQAENQLFDKW
DcTPS57      320 KGICWGRNINIADIDDTSMAFRLLRLHGYNVSQDVFRNFESKGEFFAWAGQSNQAVTGMLDLLKASQVMLPGENVLEDAKTFSYEFLRRKQANNQLLDKW

AT4G02780.1  406 IIMKDLPGEIGFALEIPWYASLPRVETRFYIDQYGGENDVWIGKTLYRMPYVNNNGYLELAKQDYNNCQAQHQLEWDIFQKWYEENRLSEWGVRRSELLE
DcTPS59      453 IITKDLQGEVEYGLDIPWYASLPRLETCFYLDHYGGEDDVWIGKTLYRMPHVNNNIYLELAKLDYAKCQTIHQQEWYHMKEWFAHSKLEQFGMNENSLLL
DcTPS25      481 IITKDLPGEVGYALDTPWYASLPRLETSFFLDQYGGEDDVWIGKTLYRMPHVNNNTYLELAKLDYAKCQTIYQLEWNQMKEWCANSNLEKFGLSETSLLL
DcTPS57      420 IIMKDLPGEVEYALDIPWYASLPRLETCFYLDQYGGEDDVWIGKTLYRMPRVNNNIYLELAKLDYAKCQTIHQLEWSHMKEWCARSNLDKLGLSENSLLL

AT4G02780.1  506 CYYLAAATIFESERSHERMVWAKSSVLVKAISSSFGE---SSDSRRSFSDQFHEYIANARRSDHHFNDRNMRLDRPGSVQAS--RLAGVLIGTLNQMSFD
DcTPS59      553 SYYLAASSLFEPELSHQRFAWAKTEALVETIGSFFENMENSVEQRKAF------------------------------------KLVGTLVGTIKQLTLN
DcTPS25      581 SYYLAASSLYEPESSNLRFAWAKTEALIETIRLYFGNIESSAEQREAFVQDYMK------------TTENLQYVSPGRYKSPRKKLLGTLVGILKQLMLD
DcTPS57      520 SYYLAASSLFEPEVSNLRYAWAKTDALVETIRSYFGNLERSVEQRKAFAQEFEN------------TSENSYFANHGRYSIE-KKLLGTLVATLKQLILD

AT4G02780.1  601 LFMSHGRDVNNLLYLSWGDWMEKWKLYG--DEGEGELMVKMIILMKNNDLTN-FFTHTHFVRLAEIINRICL---PRQYLKARRNDEKEKTIKS-----M
DcTPS59      617 AKSAHGIDIYPQLHQAWLAWLLVWQEDGNVDKARAQLLEEIINICAGRLISEEILSHPQYKTLSTITNRICHQLRVFQ--KVQNQNNCNGNTGAITTAEI
DcTPS25      669 AMVAHGIDIHQQLHQAWGMWLTTWQEEGDVDKAKAQLLEQTINICAGRLTSEEILSNPQYKNLSKITNQLCHQLGPFQYGKLNEEDSSDKEIRGITAMEI
DcTPS57      607 AKSAHGIDIYPQLHQAWTMWLLKWQEEGDVDKAKAQLLEQTINICAGRILSNEILSHPHYKTLSTITNRLCHQLSIFR--KVQNEKSRDTSRGGITTMEI

AT4G02780.1  690 EKEMGKMVELALSESDTFRDVSIT--FLDVAKAFYYFALCG-DHLQTHISKVLFQKV---
DcTPS59      715 ESDMQELVQSVFCNSPDSLDPEFKQIFFMVARTFYYTAYCDPNIINDHIGKVLFGT-RM*
DcTPS25      769 ESKMQELVKLVLCNSPDGLDPELKQIFFTLARTFYYTAYCDPITINSHISKVLFGR-RM*
DcTPS57      705 ESDMRELVRLVFCNSEDSLHPELKHIFFMVARTFYYTAYCDVDTVNGHINKVLLGSTRM*
```

Supplementary Figure S7. Amino acid sequence alignment of proteins in the carrot TPS-c sub-family and other select TPSs of this subfamily. Alignments were performed using Clustal W[43] and visualized with BoxShade (v3.21, https://embnet.vital-it.ch/software/BOX_form.html); At, *Arabidopsis thaliana*; Dc, *Daucus carota.*

```
At1g79460    1 -----------MSINLRSSGCSSPISATLERRL-----------DSEVQTRANNVSFEQTKEKIRKMLEKVELSVSAYDTSWVAMVPSPSSQNAPLFPQC
DcTPS28      1 ----------------------MTIPTLLLYLL-----------SIHILLLFERKVIEESKERIRRLFRKVELSVSAYDTAWVAMVPSPHYSHAPCFSGC
DcTPS56      1 MIYKFDFRANLPVFKYQTFSKQNTVVSYLACLFSTSKPVAFHINSILILITHLSQDIQGRKERIRSLFSKVELSVSAYDTAWVAMVPSPRHSQTPCFSNC

At1g79460   79 VKWLLDNQHEDGSWGLDNHDHQSLKKDVLSSTLASILALKKWGIGERQINKGLQFIELNSALVTDETIQKPTGFDIIFPGMIKYARDLNLTIPLGSEVVD
DcTPS28     68 VDWILENQLKDGSWGLPQQSVRLLKDD-LSSTLACVLALKRWDVGEEHINKGLRFLKSNFASVTDNNQSSPIGFDIIFPGMLEYANDLGLKLPVEQTLLN
DcTPS56    101 VDWILENQLNDGSWGLPHHNGQWLKDD-LSSTLACILALKRWGVGKEHINRGTHFLELNFGSAIDDSQPAPVGFDIIFPGMLEYATTLDLKLPLDQTTFN

At1g79460  179 DMIRKRDLDLKCDSEKFSKGREAYLAYVLEGTRNLKDWDLIVKYQRKNGSLFDSPATTAAAFTQFGNDGCLRYLCSLLQKFEAAVPSVYPFDQYARLSII
DcTPS28    167 TVLNKKAQEIKRCSERNSPESEAYLAYLSEGMGNLQNWDMVIKYQRKNGSLFNSPSTTAAYLNHIQNTGCLNYLSHLLEKFGNAVPTVYPIDIYAHLCMV
DcTPS56    200 DMLKKRDREIKRYSENQSSQSEAYLAYVSEGMGNSQNWDMIMKFQRKNGSLFNSPSTTAASLSHTQNTGCLNYLRGVLKKFGDAVPTVYPFDIYARLCMV

At1g79460  279 VTLESLGIDRDFKTEIKSILDETYRYWLRGDEEICLDLATCALAFRLLLAHGYDVSYDPLKPFAEESGFSDTLEGYVKNTFSVLELFKAAQS--YPHESA
DcTPS28    267 DTLERLGIDRHFKQEIRSVLDEAYSCWLQDDEEIFMDVDTCALAFRILRMNGYNVLSDKLTRIAKEECYLNSLGENLKDTNEALQLYRASEAIIYSNESA
DcTPS56    300 DNLDKLGIDWHFRQEIRTVLDETYSSWLQDDEEIFKDVATCALAFRILRVNGYDVDSEKLTQVAQEDYYCNSHGGHLNDTYEALELYRASQNIIYPNETA

At1g79460  377 LKKQCCWTKQYLEMELSSWVKTSVRDKYLK---KEVEDALAFPSYASLERSDHRRKILNGSAVENTRVTKTSYRLHNICTSDILKLAVDDFNFCQSIHRE
DcTPS28    367 LEKQNSWSNRFLEHKLSN--GSVHLDRCARIIFQEVHDALKFPFHSNLERMVNRRNI-EQYEADSIKILKTSYSSPNISNAEYVRLAVEDFNVCQSIQQK
DcTPS56    400 LEKQNSWSKNFLQRKLNN--RSVHSDKYTSAMFQEVEYALKFPYYATLERVVHRRSI-EQYNTGNLRILKTLCLSENISNTEFPRFAMEDFNTSQSIYQE

At1g79460  474 EMERLDRWIVENRLQELKFARQKLAYCYFSGAATLFSPELSDARISWAKGGVLTTVVDDFFDVGGSKEELENLIHLVEKWDLNGVPEYSSEHVEIIFSVL
DcTPS28    464 ELKLLESWVIESSLDKLKFARQKTAYCYFSAAATLFSPELSDARMAWAKNGILTTVVDDFFDVGGSIEELLNLIQLVEKWDVNEETECCSEHVRIIFSAL
DcTPS56    497 ELKLLESWTVDNKLKTLEFAREKNVYCYFCAAAMIFPPELHEARITWAKYSILTAKVDDFFDNGGSMEELLNLIQLFKKWNVDVHTECCSENVRILFLAL

At1g79460  574 RDTILETGDKAFTYQGRNVTHHIVKIWLDLLKSMLREAEWSSDKSTPSLEDYMENAYISFALGPIVLPATYLIGPPLPEKTVDSHQYNQLYKLVSTMGRL
DcTPS28    564 HHTICEIGESAFKRQAWHVTTHIIEIWLELLNSMLKEAEWTRDSYVPKLDEYMSNGFISFALGPIVLPTVYLIGPELSENVVQNGELRSLFKLMSTCGRL
DcTPS56    597 RSTICETVDWASKWQGRNVTNHMIEIWMEVLDTMLKEAEWARGTYVPKMDEYIENGYVSFALGPIVLPTIYLVGPQLSESVVGSDELRNLFKLMSMCGRL

At1g79460  674 LNDIQGFKRESAEGKLNAVSLHMKHERDNRSKEVIIESMKGLAERKREELHKLVLEEKGSVVPRECKEAFLKMSKVLNLFYRKDDGFTSNDLMSLVKSVI
DcTPS28    664 LNDIQSFKRESKEGKLNAVSLYMIHT-DATTEEDAINEIKCAIEINRRELLQLVLQEKDSVVPRACKDLFWKMCRVVHQFYIKDDGFTSEDMFGAVKDIL
DcTPS56    697 LNDIQSYKRESKQGKLNYVPLHMINT-DSSSEEDAVGEIKRIIDDQRRELQRLVVEEKESVVPRVCKDLFWKMSRVVHLFYMNDDGFTLENMFGAVKDVL

At1g79460  774 YEPVSLQEESLT
DcTPS28    763 YKPITRV*----
DcTPS56    796 HKPISSDLK*--
```

Supplementary Figure S8. Amino acid sequence alignment of proteins in the carrot TPS-e sub-family and other select TPSs of this subfamily. Alignments were performed using Clustal W[43] and visualized with BoxShade (v3.21, https://embnet.vital-it.ch/software/BOX_form.html); At, *Arabidopsis thaliana*; Dc, *Daucus carota.*

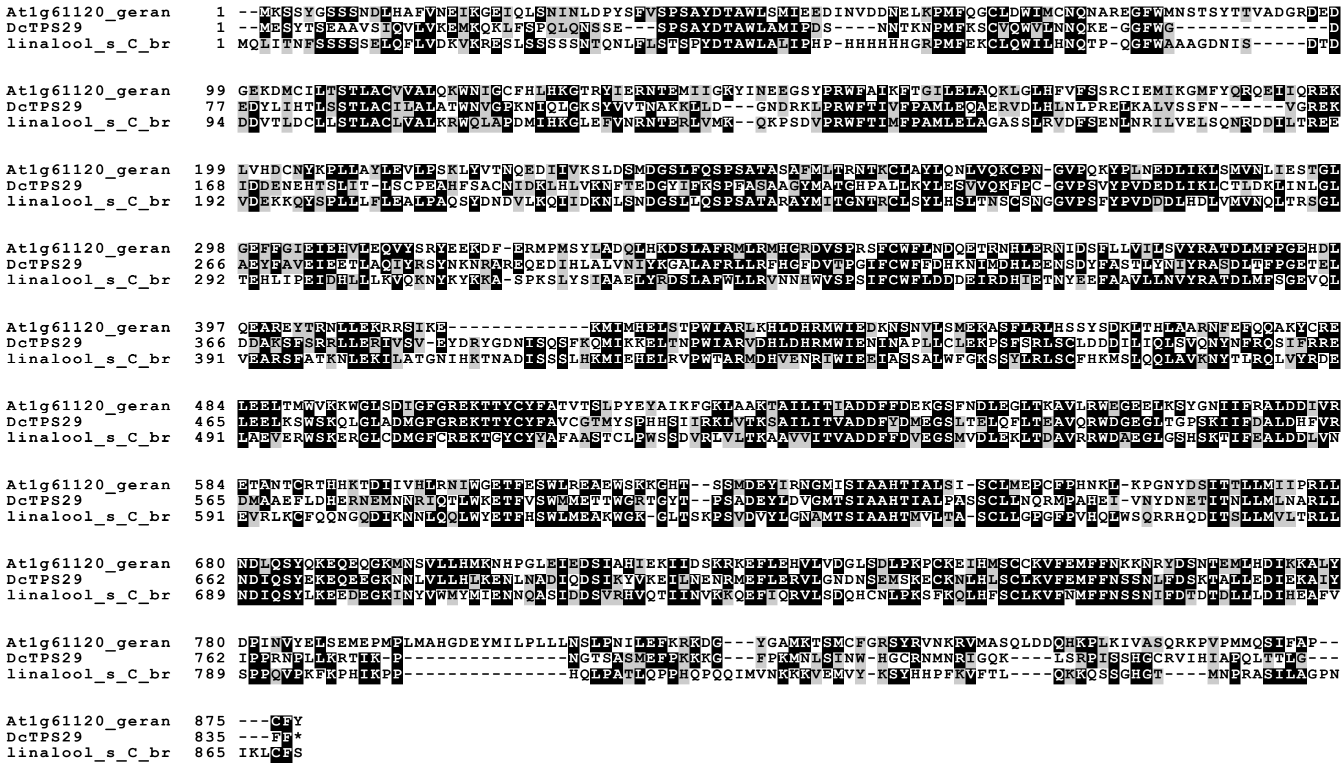

Supplementary Figure S9. Amino acid sequence alignment of proteins in the carrot TPS-f sub-family and other select TPSs of this subfamily. Alignments were performed using Clustal W[43] and visualized with BoxShade (v3.21, https://embnet.vital-it.ch/software/BOX_form.html); At, *Arabidopsis thaliana*; Cb, *Clarkia breweri*; Dc, *Daucus carota.*

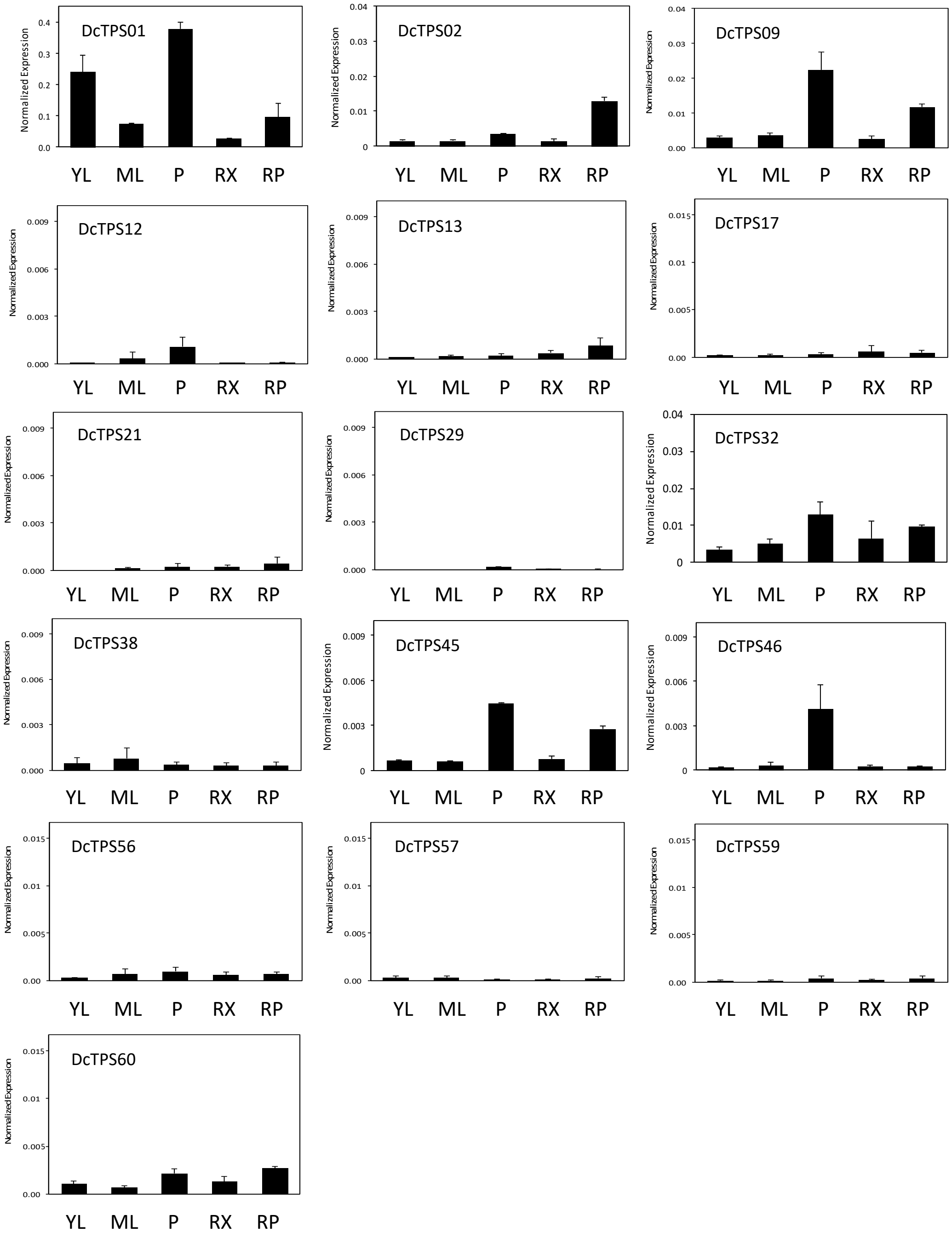

Supplementary Figure S10. qRT-PCR analysis of transcript abundance of TPS genes that were identified previously[17] or not functionally characterized in this study. Relative expression levels across tissues for each gene were calculated using the $\Delta\Delta C_T$ standard method normalized to expression of actin. Amplifications were performed in biological and technical triplicate and error bars indicate standard deviation from the mean. YL: young leaf, ML: mature leaf, P: petiole, RX: root xylem and RP: root phloem.

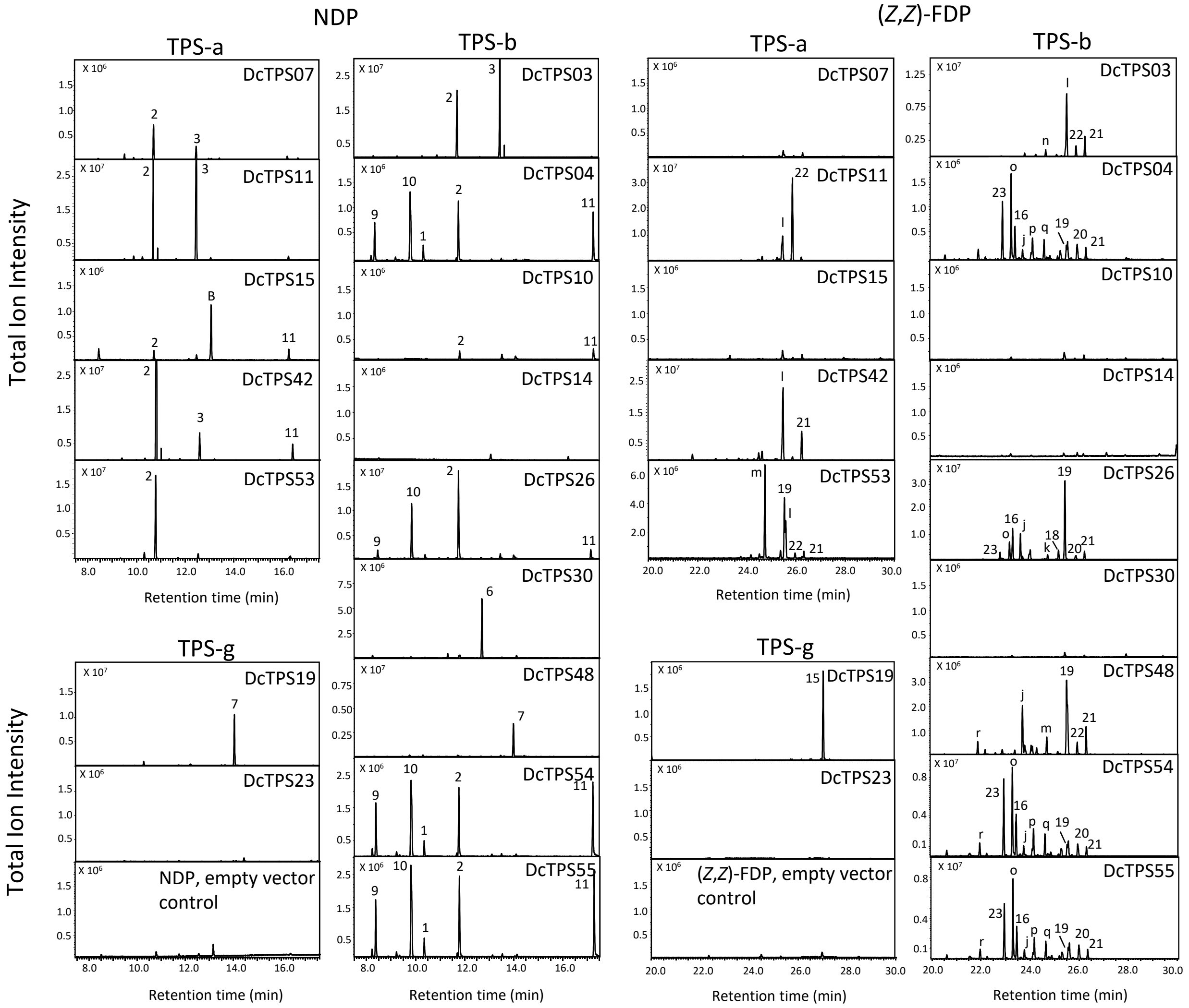

Supplementary Figure S11. SPME-GC-MS analysis of terpene products from assays with recombinant TPS enzymes. Partially purified proteins were incubated in the presence of NDP or (*Z*,*Z*)-FDP. Numbering of compounds matches that of Figure 4. 1: β-myrcene*, 2: limonene*, 3: α-terpinolene*, 6: γ-terpinene*, 7: linalool*, 9: α-pinene*, 10: sabinene*, 11: α-terpineol*, 15: nerolidol*, 16: (*E*)-α-bergamotene*, 18: (*Z*)-α-bisabolene*, 19: β-bisabolene*, 20: sesquiphellandrene*, 21: (*E*)-α-bisabolene*, 22: (*E*)-γ-bisabolene*, 23: (*Z*)-α-bergamotene*. * indicates compounds that were identified with authentic standards or by comparison with compounds of Opopanax oil. Mass spectra of these compounds are shown in Supplementary Figure S12. Lower case letters indicate additional terpene compounds with identification confidence levels <90%. Mass spectra of these compounds are depicted in Supplementary Figure S13. Note that retention times of products obtained from TPS-a enzymes with NDP are shifted by 1 min due to difference in column condition. B; SPME fiber-related background occurring in some assays.

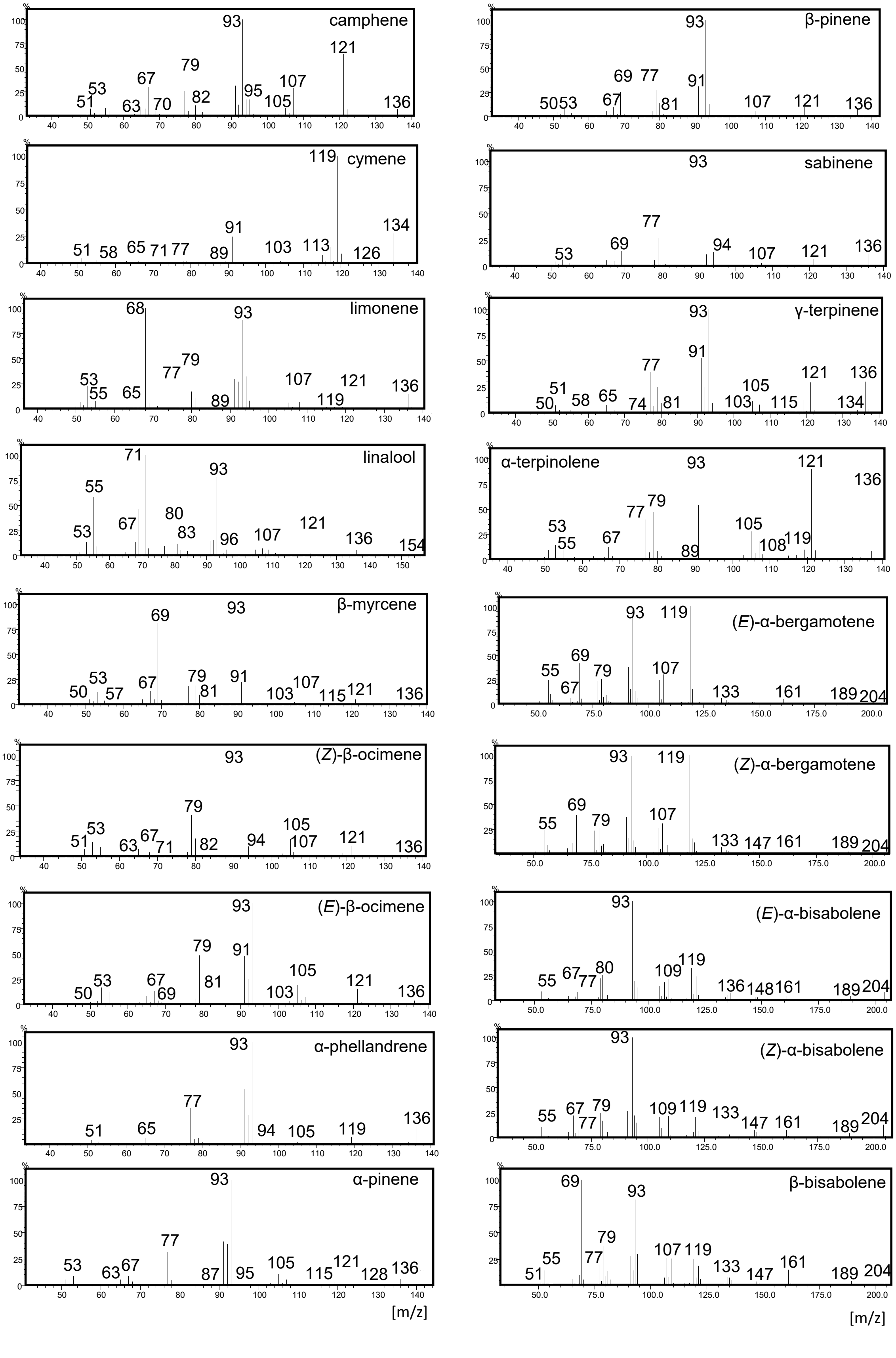

Supplementary Figure S12. Mass spectra of enzymatic products identified by comparison to authentic standards and components of Opopanax oil.

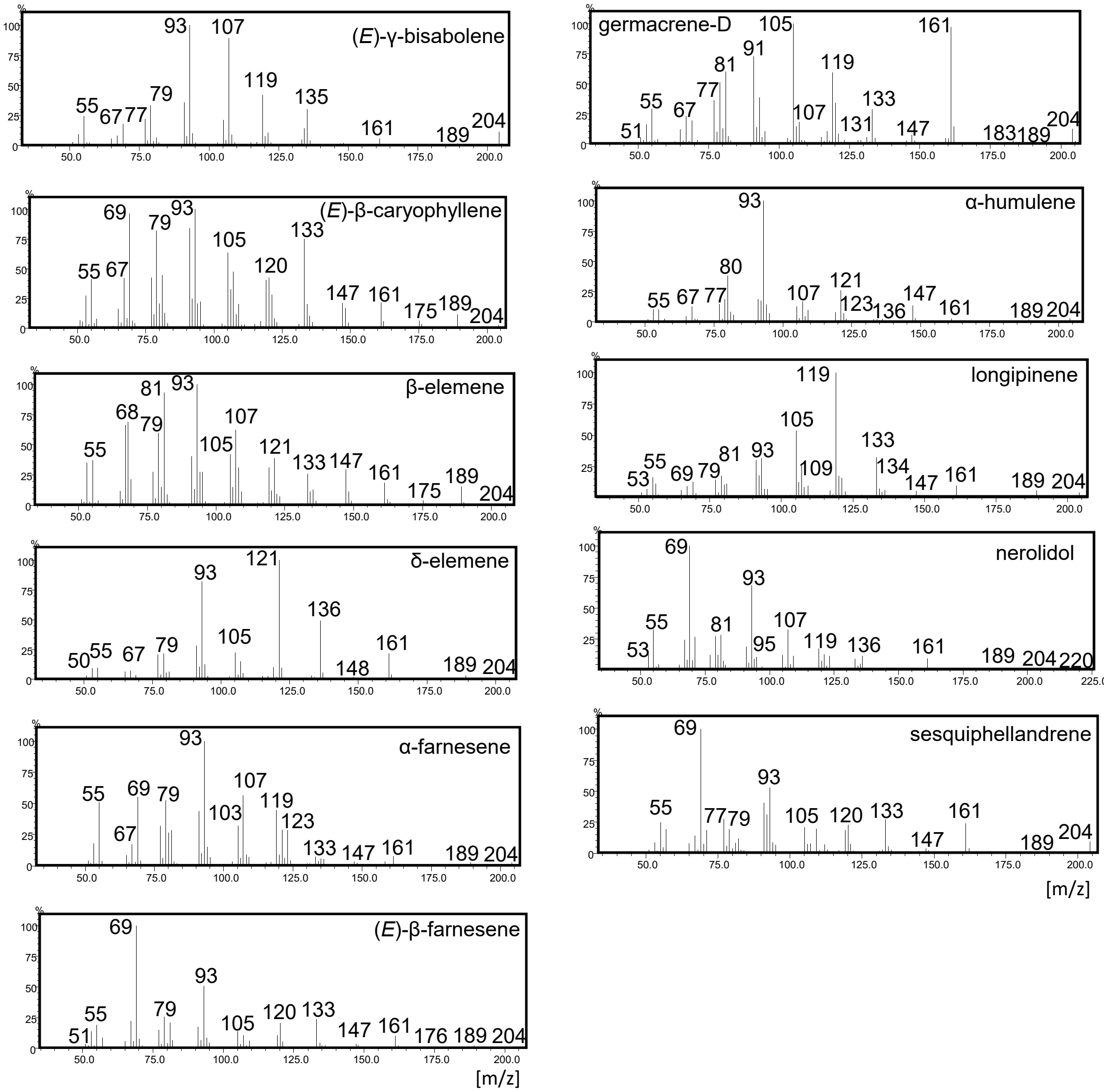

Supplementary Figure S12 continued.

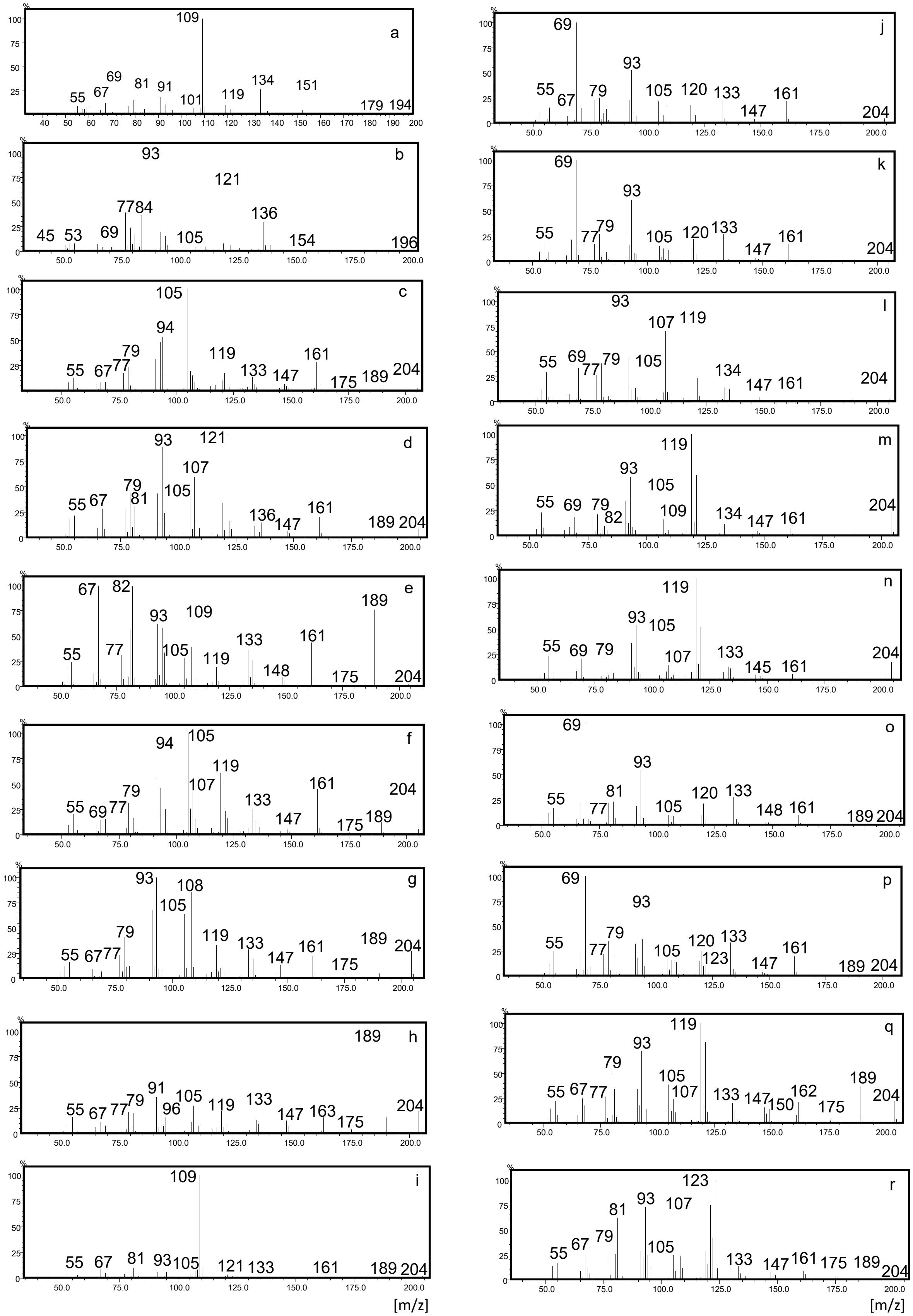

Supplementary Figure S13. Mass spectra of enzymatic sesquiterpene products with identification confidence levels <90%. The letters refer to compound peaks shown in Figure 4 and Supplementary Figure S11. Compounds j, n, and o are putative farnesene isomers. Compound k is a putative bisabolene isomer.

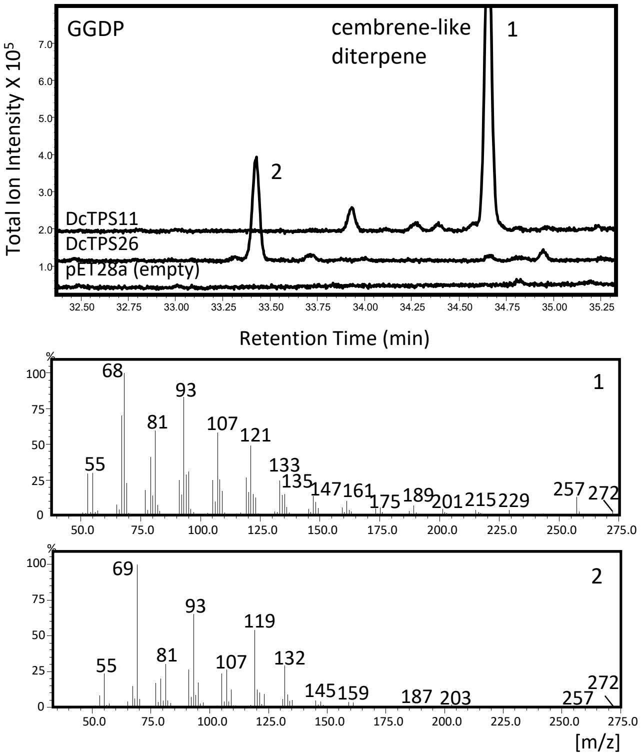

Supplementary Figure S14. SPME-GC-MS analysis of enzyme assays with GGDP and partially purified recombinant *Dc*TPS11 and *Dc*TPS26 protein or empty vector control. Assays were incubated for 5 min in the presence of a SPME fiber prior to thermal desorption. Putative compound identification is based on comparisons to reference libraries (NIST and Wiley). Mass spectra of putative diterpene products 1 and 2 are presented below.

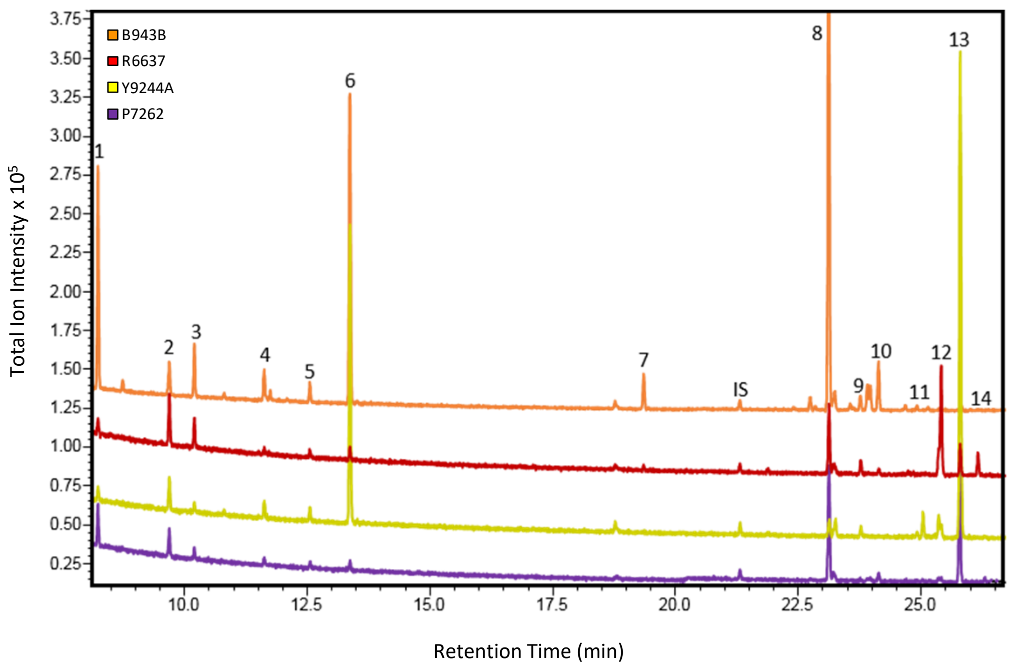

Supplementary Figure S15. GC-MS analysis of hexane extracts from roots of 11-week old, field grown colored carrot cultivars. 1: α-pinene*, 2: β-pinene*, 3: β-myrcene*, 4: limonene*, 5: γ-terpinene*, 6: α-terpinolene*, 7: bornyl acetate, 8: (*E*)-β-caryophyllene*, 9: (*E*)-β-farnesene*, 10: α-humulene*, 11: β-bisabolene*, 12: Putative bisabolene isomer, 13: (*E*)-γ-bisabolene*, 14: (*E*)-α-bisabolene*, IS: internal standard 1-bromodecane. *indicates compounds that were identified with authentic standards or by comparison with compounds of Opopanax oil. Other labeled compounds were identified by library comparison only. Purple (cv. P7262), Red (cv. R6637), Yellow (cv. Y9244A) and Orange (cv. B493B).

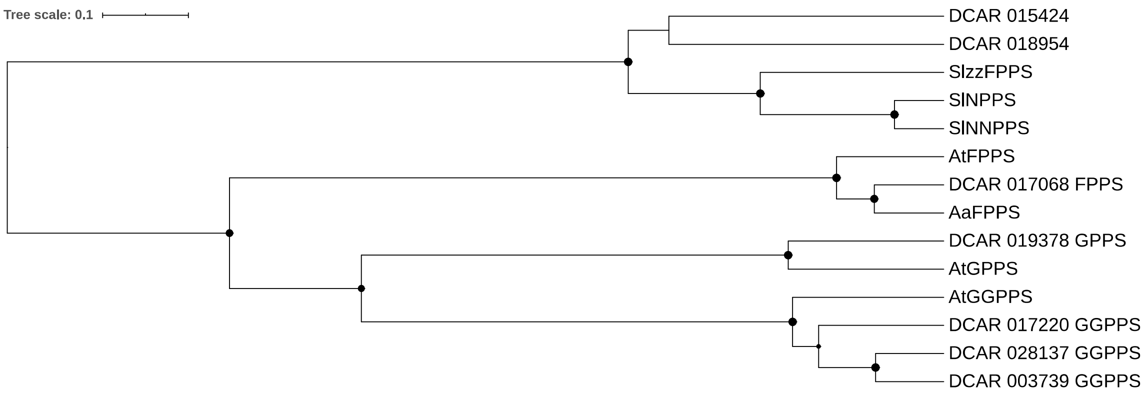

Supplementary Figure S16. Maximum-likelihood phylogenetic tree of putative carrot (DCAR) *trans*- and *cis*-IDSs and known *trans*- and *cis*-IDS from other plant species. Circles indicate bootstrap support of > 80% where bootstrap replicates = 500. At, *Arabidopsis thaliana*; Aa, *Artemisia annua*; Sl, *Solanum lycopersicum.* GPPS, geranyl diphosphate synthase; FPPS, farnesyl diphosphate synthase; GGPPS, geranylgeranyl diphosphate synthase; NPPS, neryl diphosphate synthase; NNPPS, nerylneryl diphosphate synthase.
